# Density-functional tight-binding for phosphine-stabilized nanoscale gold clusters†

**DOI:** 10.1039/d0sc04514d

**Published:** 2020-11-02

**Authors:** Van Quan Vuong, Jenica Marie L. Madridejos, Bálint Aradi, Bobby G. Sumpter, Gregory F. Metha, Stephan Irle

**Affiliations:** Bredesen Center for Interdisciplinary Research and Graduate Education, University of Tennessee Knoxville TN USA; Department of Chemistry, The University of Adelaide South Australia 5005 Australia greg.metha@adelaide.edu.au; Bremen Center for Computational Materials Science, University of Bremen Bremen Germany; Center for Nanophase Materials Sciences, Oak Ridge National Laboratory Oak Ridge TN USA; Computational Sciences and Engineering Division, Oak Ridge National Laboratory Oak Ridge TN USA irles@ornl.gov

## Abstract

We report a parameterization of the second-order density-functional tight-binding (DFTB2) method for the quantum chemical simulation of phosphine-ligated nanoscale gold clusters, metalloids, and gold surfaces. Our parameterization extends the previously released DFTB2 “auorg” parameter set by connecting it to the electronic parameter of phosphorus in the “mio” parameter set. Although this connection could technically simply be accomplished by creating only the required additional Au–P repulsive potential, we found that the Au 6p and P 3d virtual atomic orbital energy levels exert a strong influence on the overall performance of the combined parameter set. Our optimized parameters are validated against density functional theory (DFT) geometries, ligand binding and cluster isomerization energies, ligand dissociation potential energy curves, and molecular orbital energies for relevant phosphine-ligated Au_*n*_ clusters (*n* = 2–70), as well as selected experimental X-ray structures from the Cambridge Structural Database. In addition, we validate DFTB simulated far-IR spectra for several phosphine- and thiolate-ligated gold clusters against experimental and DFT spectra. The transferability of the parameter set is evaluated using DFT and DFTB potential energy surfaces resulting from the chemisorption of a PH_3_ molecule on the gold (111) surface. To demonstrate the potential of the DFTB method for quantum chemical simulations of metalloid gold clusters that are challenging for traditional DFT calculations, we report the predicted molecular geometry, electronic structure, ligand binding energy, and IR spectrum of Au_108_S_24_(PPh_3_)_16_.

## Abstract 2

We report a parameterization of the density-functional tight-binding (DFTB) method for the accurate prediction of molecular, electronic and vibrational structure of phosphine-ligated nanoscale gold clusters, metalloids, and gold surfaces.

## Graphical abstract

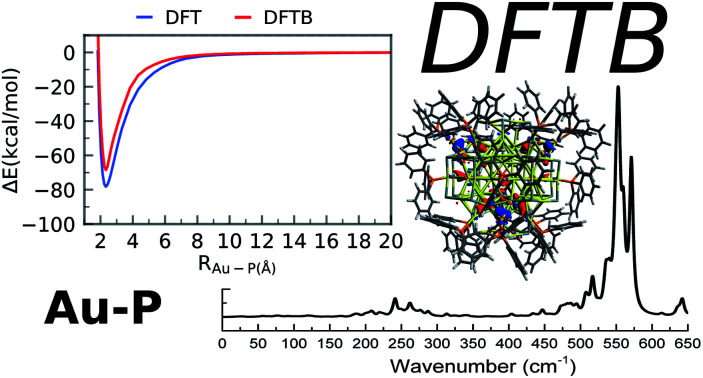

## 1 Introduction

Atomically precise ligated gold clusters of nanometer dimension receive continued attention due to their unique catalytic properties^1–3^ and well-defined discrete electronic energy levels^4^ that potentially offer greater flexibility and control over the more metal-like states of the corresponding bare gold nanoparticles and complexes.^3,5^ Nanoscale gold clusters have shown high catalytic activity and selectivity for certain reactions at low temperature, such as the oxidation of carbon monoxide, propene and alcohols, or the hydrogenation of acetylene.^3,6–8^ Commonly chosen “capping ligands” employed to stabilize atomically precise nanoscale gold clusters are typically thiolates and phosphines, which prevent aggregation, coalescence and unlimited growth during synthesis.^9^ Post-treatment is usually required to remove some (or all) of the ligands to allow interaction with the substrate or reactants.^10–14^ To achieve control over such complex catalytic systems it is vitally important to understand the relationship between molecular and electronic structure, often studied by a combination of experimental and theoretical approaches.^15,16^ In addition, a better knowledge of the energetics associated with ligand removal is required to identify how post-treatment can be done without inducing concomitant side-effects such as agglomeration. Density functional theory (DFT) methods are most often employed in theoretical investigations, as they are capable to accurately describe electronic, geometrical, and vibrational structure of gold clusters and nanoparticles.^16–29^ In particular, the DFT-based simulation of IR, Raman, and UV-vis spectra has been achieved for a range of gold clusters, thus providing useful theoretical fingerprints to distinguish between bonding arrangements and orientations between gold atoms and ligands, and ligand–ligand interactions within clusters.^23,30–34^

Unfortunately, DFT calculations can become prohibitively expensive with system size,^35^ and routine theoretical investigations are limited to moderate system sizes. One way of reducing the computational cost is to entirely neglect the ligands and only perform DFT calculations on the gold cores. An alternative way is to employ simplified ligand models where *e.g.* triphenylphosphine (PPh_3_) is replaced by phosphine (PH_3_) or trimethylphosphine (PMe_3_).^17,18,22,25,26,28,29,36^ This, as with the complete ligand removal approach, has the additional benefit that conformational searching is simplified, as the torsions of the three phenyl groups per ligand give rise to a large number of local minima with similar energies. Nevertheless, it is well known that the electronic effects of the larger ligands are different from those of the smaller ones, for instance inductive effects,^36–39^ and a computational truncation of the ligands will influence the chemistry and therefore description of the catalytic properties in calculations. Integrated schemes such as ONIOM^40^ may be used to capture such electronic effects in calculations on the untruncated “real” systems; however, the choice for high and low levels of theory and the definition of the interface between them is not straightforward. A rigorous ONIOM study requires benchmarks of the selected methods against a high-level calculation for the “real” system,^41^ which is often computationally unfeasible. In practice therefore, integrated methods are often difficult to employ in the context of ligated nanoscale metal clusters. Besides the computational effort related to the proper modeling of the ligands, the size of the metal cluster itself can become problematic for conventional DFT studies, severely impacting the size range of gold clusters to be investigated for property control and fine-tuning. Exacerbating this problem is the recent emergence of larger, so called metalloid, clusters such as Au_108_S_24_(PPh_3_)_16_.^25^ To make matters even worse, interactions of deposited gold clusters with substrate surfaces such as SiO_2_ and TiO_2_ may play an important role in the catalytic reaction,^10–14,42^ which further increases the computational expense of DFT studies. A recent review on connections between theory and experiment for gold nanoclusters has thus posed the question as to how theoretical calculations can be expanded to treat larger sizes and length scales.^15^

Semi-empirical electronic structure methods offer the capability to simulate large systems with explicit inclusion of electronic structure by introducing empirical parameters and methodological approximations to rigorous *ab initio* or first principles methods.^43–48^ Among them, density-functional tight-binding (DFTB),^45,49–53^ an approximation to DFT formulated in the framework of non-orthogonal tight binding, has emerged as one of the most accurate, and potentially versatile choices. DFTB is capable of simulating systems containing many thousands of atoms with an accuracy comparable to traditional DFT methods.^46,54,55^ As the DFTB method takes advantage of the two-center approximation, tabulated Hamiltonian and overlap integrals within the Slater–Koster scheme,^51,56^ it is two to three orders of magnitudes faster than DFT. In order to apply DFTB into the theoretical study of ligated gold clusters, it is necessary to provide accurate parameters for all binary chemical element interactions, most notably Au–S and Au–P. The Au–S interaction in combination with the “mio” parameter set^51,52,57^ was included in the “auorg” parameter set for gold–thiolates clusters,^58,59^ but the parameters for the Au–P interaction have not been developed.

In this work, we report a parameterization of the Au–P interactions for the second-order DFTB (DFTB2) method for the sake of compatibility with the previously developed parameters. The accuracy of these new DFTB parameters is probed by comparing the corresponding properties against DFT-calculated values and available experimental results in terms of: (1) root mean square deviations of optimized geometries, (2) energetic properties, *i.e.* ligand binding energies, relative isomer energies, and ligand dissociation energy profiles, (3) electronic structure, (4) vibrational normal modes of Au–P containing clusters, and (5) the adsorption of a PH_3_ molecule on the gold (111) surface. Finally, we present DFTB-based predictions for structural, energetic, and vibrational properties of the recent experimentally reported metalloid gold cluster Au_108_S_24_(PPh_3_)_16_.^25^

## 2 Methodology and computational details

### 2.1 Brief overview of DFTB2

A comprehensive review of DFTB methods can be found elsewhere^60^ and will not be repeated here. In this work, we only focus on generating parameters for the DFTB2 method, which is also referred to as self-consistent-charge (SCC)-DFTB.^51^ The DFTB2 total energy can be viewed as a 2nd order Taylor expansion of the Kohn–Sham energy with respect to a reference initial electron density *ρ*_0_ and electron density fluctuations Δ*ρ*1
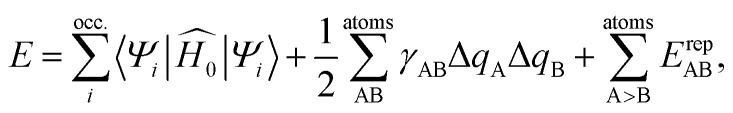
where 
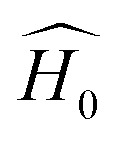
 is the initial Hamiltonian constructed from the superposition of neutral atomic densities in a two-center approximation,^49^ and the |*Ψ*_*i*_〉 are occupied valence molecular orbitals (MOs), expanded as a linear combination of optimized pseudo-atomic orbitals |*ϕ*_μ_〉. The optimization of these pseudo-atomic valence orbitals and orbital densities for a given chemical element constitutes the determination of the electronic parameters. Δ*q*_A_ is a point charge^61^ on atom A, and *γ*_AB_Δ*q*_A_Δ*q*_B_ represents the Coulomb interaction energy between the two point charges;^51^ when A = B, *γ*_AA_ is the chemical hardness or second derivative of the total energy with respect to the charge on atom A. *γ*_AB_ is defined as2
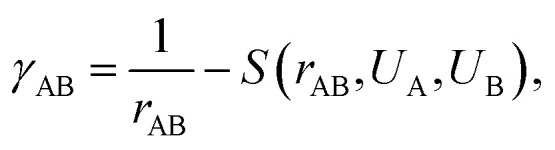
where *S* is an exponentially decaying short-range function that depends on the distance between the two atoms *r*_AB_ = |*r*_A_ − *r*_B_| and their chemical hardness, given in form of the so-called Hubbard parameter *U*. The latter is calculated prior to molecular DFTB calculations for each chemical element using the DFT method, typically employing the Perdew–Burke–Ernzerhof (PBE) functional.^62^

In the framework of the two-center approximation, the Hamiltonian integrals 
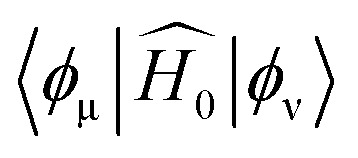
 and the overlap integrals 〈*ϕ*_μ_|*ϕ*_ν_〉 are pre-tabulated for each chemical element pair. As in all tight binding approaches, the DFTB2 total energy consists of an electronic energy, which is the sum of the first two terms in eqn (1), and a summation over all unique repulsive potentials between two atoms *E*^rep^_AB_. The latter are formulated as a two-center term that depends only on the chemical element type of atoms A and B and their interatomic distance *r*_AB_.^60^ In principle, these pairwise repulsive potentials can be pre-calculated analytically from DFT for diatomic molecules. However, it was found that the performance of such DFT-based repulsive potentials is usually not sufficiently accurate for general purposes.^59^ Therefore, in practice, one computes DFT- or wave function theory (WFT)-based reference relative energies for model systems that contain various bond lengths of the chemical element pair in question, and fits the total repulsive energy such as to minimize the difference between reference and resulting DFTB relative energies. For this purpose, the repulsive potentials are approximated by a combination of exponential and spline functions3
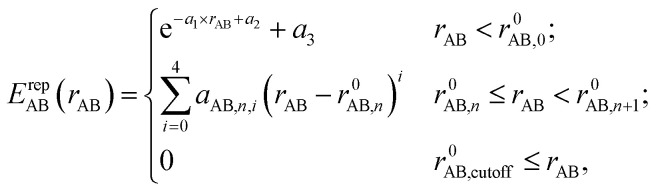
where “*r*^0^_AB,*n*_” is a spline knot at the *n*^th^ interval, and the “*a*_AB,*n*_” are the polynomial spline coefficients. These variables are considered the free empirical parameters and optimized either by hand or automatically^63–65^ to minimize the difference between reference and DFTB relative energy for a series of training systems. In addition to relative energies, repulsive potentials can also be optimized to fit molecular geometries by minimizing the difference between the reference DFT or WFT energy gradient and the DFTB energy gradient for a set of equilibrium and non-equilibrium geometries in the training set. The possible constraints on the repulsive potentials include a cutoff radius, a limit on the number of allowable extrema, and a continuity requirement up to a given derivative order.

### 2.2 Gold–phosphorus parameterization

As indicated above, there are two groups of DFTB2 parameters that need to be determined: (1) the electronic parameters, and (2) the repulsive potentials for pairs of chemical elements. The electronic parameters are comprised of the radii *r*^wf^ used in the definition of atomic confinement potentials for generating pseudo-atomic orbitals |*ϕ*_μ_〉 as well as the confinement radii *r*^dens^ for the atomic density; additional electronic parameters are the atomic orbital energies and the atomic Hubbard parameters *U* for each chemical elements.^51^ While the AO energies and the Hubbard parameters *U* are normally taken from DFT calculations of the free atom, the other electronic parameters and pairwise repulsive potentials are subject to optimization with the goal to reproduce certain desired properties; for instance, electronic band structure, atomization energies, reaction energies, and geometries (energy gradients or atomic forces). In order to parameterize the Au and P interactions for DFTB2, we adopted the Au electronic parameters from the “auorg” set published by Fihey *et al.* (referred to as auorg^*α*^),^58^ and a modified version of “auorg” by Oliveira *et al.* (referred to as auorg^*χ*^).^59^ The difference between these two parameter sets lies in the Au 6p-orbital energy; in the auorg^*α*^ set it was taken as the true PBE orbital energy, while in the auorg^*χ*^ set it was empirically shifted upward by ≈+0.0279 hartree. The main purpose for this orbital energy shift was to obtain improved values for cohesive energies of pure gold nanoclusters with respect to PBE.^59^ Following the work of “auorg”, only 5d and 6 s valence electrons are considered, in total 11 valence electrons per Au atom. The parameters of the other elements in the auorg^*α*^ and auorg^*χ*^ sets were taken from the “mio” parameter set.^51,52^ Consequently, we adopted the electronic parameters for P taken from the “mio” parameter set as well. It is important to mention that the 3d orbital energy of the P atom had been shifted by +0.5 hartree from its PBE-calculated value of 0.02044 hartree, according to Gaus *et al.* to reduce the overbinding in phosphate compounds, as the shift had also been adopted in the “3ob” parameter set for the DFTB3 method.^52,66^ However, in the case of Au–P interactions, such a drastic shift of the P 3d virtual orbital energy introduces significant underbinding. We therefore decided to investigate the effect of the P 3d orbital energy level in detail. We systematically increased the value of the P 3d orbital energy by increments of 0.1 hartree from its PBE computed and the “mio” shifted value, and used our genetic algorithm (GA) optimization tool^65^ to automatically generate repulsive potentials for the Au–P interaction with these orbital energy shifts. In this way we tested the performance of the resulting Au–P parameterization with special consideration of geometries and binding energies for the adsorption of a PH_3_ on Au (111). An energy value of 0.12044 hartree was determined as the optimal compromise for the P 3d orbital. We refer to Table S1 in the ESI† for the performance of DFTB2/mio with different P 3d orbital energies for a selected test set of chemical reactions involving H, C, N, O, P, and S containing compounds.

We decided to introduce a nomenclature for denoting the different choices of Au 6p and P 3d orbital energies in our parameters. Following Oliveira *et al.*^59^ we denote the original PBE Au 6p energy value by ‘*α*’ and its modified value by ‘*χ*’. The shifted “mio” P 3d energy will leave these notations unchanged, while the use of our new optimized P 3d orbital energy value of 0.12044 hartree will be denoted with a prime, ‘′’. Hence, auorg^*α*^ coupled with the original “mio” P is unchanged, while the auorg^*α*^ and auorg^*χ*^ coupled with the new P 3d-orbital energy are referred to as auorg^*α*′^ and auorg^*χ*′^, respectively. Because auorg^*χ*^ exhibits the largest underestimation of the Au–P electronic binding energy (both Au 6p and P 3d virtual orbital energies are shifted upwards from PBE values), we did not generate the Au–P repulsive potential for this orbital energy combination. Table 1 summarizes the nomenclature of the three different parameter sets and the relationship with their employed Au and P virtual atomic orbital energies.

**Table:** Energies in hartree for Au 6p and P 3d virtual atomic orbitals in different parameter sets

	auorg^*α*^	auorg^*α*′^	auorg^*χ*′^
*ε* ^6p^_Au_	−0.02786	−0.02786	−0.00001
*ε* ^3d^_P_	0.52044	0.12044	0.12044

The Au–P repulsive potentials were optimized on the basis of shell-resolved self-consistent charge electronic energies to fit ligand binding energies and forces for the training set listed in Table 2 using our in-house genetic algorithm (GA)-based parameterization tool.^65^ After several preliminary tests, we employed a cutoff radius of 4 Å and 5 spline knots (the maximum for *n* in eqn (3) was equal to 5), one allowable extremum, and a continuity requirement up to the third derivative. For the GA optimization, population sizes of 3000 and 5000 generations were employed with two-point crossover and random mutation rates of 0.9 and 0.2, respectively. The GA was used to minimize a scoring function *F*^score^ defined as the fitness of the parameter sets with respect to DFTB values and reference data according to the formula4
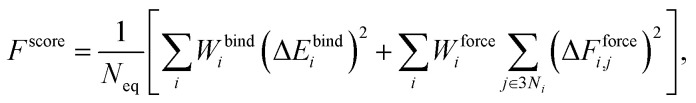
where Δ*E*^bind^ = *E*^bind^_eDFTB_ − *E*^bind^_ref_ is the deviation in ligand binding energies, Δ*F*^force^ = *F*^force^_eDFTB_ − *F*^force^_ref_ is the deviation in forces, *W*^bind^_*i*_ and *W*^force^_*i*_ are weight factors of the *i*^th^ binding energy and the force of the *i*^th^ structure, respectively, *N*_eq_ is the number of fitting data points, *N*_*i*_ is the number of atoms in the *i*^th^ compound, and eDFTB stands for the DFTB energy without the repulsive potential term. In the training, we empirically shifted the reference ligand binding energies of PH_3_ by −24 kcal mol^−1^ for auorg^*α*′^ and −22 kcal mol^−1^ for the auorg^*χ*′^ to better reproduce ligand binding energies of larger ligands and the adsorption of PH_3_ on the Au (111) surface. Surprisingly, auorg^*α*′^ and auorg^*χ*′^ optimized Au–P repulsive potentials are almost identical, see Fig. S1 in the ESI.† In principle, the Au–P repulsive potential for auorg^*α*^ can also be optimized in a similar way to maximize its performance, however, for the sake of simplicity and transferability we decided to use the same Au–P repulsive potential for auorg^*α*′^ and auorg^*α*^.

Training set for Au–P repulsive potential fitting and weights for each complex used in the fitting with eqn (4)

**Table:** 

Forces (equilibrium geometries)		
Complexes	Structures	Weights
Au_1_–PH_2_	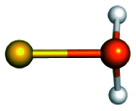	1.0
[Au_1_–PH_3_]^+^	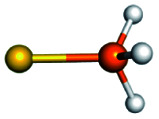	1.0
[Au_3_–PH_3_]^+^	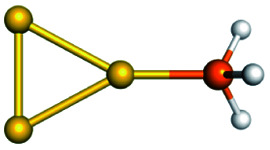	1.0
[Au_4_–PH_2_]^+^	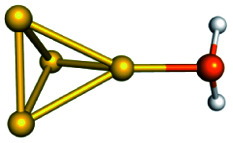	1.0
[Au_4_–PH_3_]^2+^	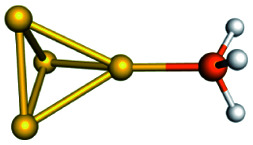	1.0
[Au_6_–PH_3_]^2+^	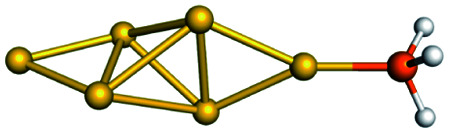	1.0
[Au_6_(planar)–PH_3_]^2+^	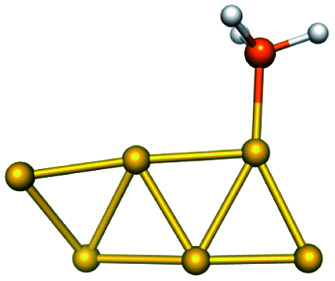	1.0

**Table:** 

Forces (distorted geometries)		
Complexes	Δ*R*_Au–P_ (Å)	Weights
[Au_1_–PH_3_]^+^	−0.3	0.5
[Au_1_–PH_3_]^+^	−0.2	0.5
[Au_1_–PH_3_]^+^	−0.1	0.5
[Au_3_–PH_3_]^+^	+0.1	0.5
[Au_3_–PH_3_]^+^	+0.2	0.3
[Au_3_–PH_3_]^+^	+0.3	0.1

**Table:** 

Ligand binding energies		
Reactions	Δ*E* (kcal mol^−1^)	Weights
[Au_3_]^+^ + PH_3_ ⇒ [Au_3_–PH_3_]^+^	−55.53	1.0
[Au_4_]^2+^ + PH_3_ ⇒ [Au_4_–PH_3_]^2+^	−76.27	1.0
[Au_6_]^2+^ + PH_3_ ⇒ [Au_6_–PH_3_]^2+^	−65.01	1.0
[Au_6_(planar)]^2+^ + PH_3_ ⇒ [Au_6_(planar)–PH_3_]^2+^	−64.58	1.0

### 2.3 Computational details

In the original parameterization of the auorg set,^58^ the generalized gradient approximation (GGA) PBE density functional was selected to generate reference geometries and cluster binding energies. In this work, we opted for the TPSS density functional^67^ because it was noted by Kepp^68^ and Goel *et al.*^69^ that this *meta*-GGA functional reproduces experimental or high-level theory bond energies and ligand–gold distances better than the standard GGA PBE functional. For the training set of small clusters in Table 2, the reference data were computed by the TPSS in combination with Ahlrich's triple-zeta valence polarized basis set (def2-TZVP).^70^ No dispersion correction was employed in the calculations generating the training set in order to avoid complications originating from a possible convolution of DFTB repulsive energy terms and the long-distance dispersion term.

In order to benchmark the accuracy of the new parameters, ligand binding energies and optimized geometries were compared to their TPSS counterparts for various complexes of PH_3_, PMe_3_, PPh_3_ and small- to moderate-sized gold clusters. For the larger complexes, which are listed in Table 3, the experimental structures from the Cambridge Structural Database (CSD) were used as the reference geometries. The different phosphorus-containing ligands considered in this test set are triphenylphosphine (PPh_3_), 1,1-bis(diphenylphosphino) propane (dppm), 1,3-bis(diphenylphosphino) propane (dppp), methyldiphenylphosphine (PMePh_2_), tris(2-(diphenylphosphino) ethyl) phosphine (PP_3_) and 1,8-bis(diphenylphosphino) octane (dppo). Additionally, there are thiol-containing ligands in some clusters comprised of reduced S_2_^−^ and *meta*-methylbenzenethiol (*m*-MBT). For these test sets, the reference calculations were performed at the TPSS/def2-SVP^70^ level of theory. Here we included the empirical D3 dispersion contribution^71,72^ in both DFT and DFTB2 calculations.^73^ The D3 dispersion correction was used to improve description of ligand–ligand interactions and ligand effects which are deemed to be important factors on the structural, electronic, and vibrational properties of ligated gold clusters.^23^ In the DFT calculations we employed an effective core potential (ECP)^74^ for the Au atoms. In order to accelerate the DFT calculations, we employed the resolution-of-the-identity (RI) approximation with the corresponding auxiliary basis sets.^75^ All non-periodic DFT calculations were carried out using the implementation of the ORCA code.^76^ DFTB2 single point energy and geometry optimization calculations were performed with the DFTB+ code.^77^ All DFTB calculations were carried out with the shell-resolved SCC option “OrbitallyResolvedSCC = Yes”. The auorg parameter was designed such that this option mostly affects the charge distribution on the gold atoms themselves and their interactions with the other elements.

**Table:** Experimental crystal structures taken from the CSD database for the test set

Complexes	CSD codes
[Au_6_(dppp)_4_]^2+^	BOTSOS^78^
[Au_6_(PPh_3_)_6_]^2+^	CATPAO10 (ref. 79)
[Au_7_(PPh_3_)_7_]^+^	BIXZAK^80^
[Au_8_(PPh_3_)_7_]^2+^	BASWUN10 (ref. 80)
[Au_8_(PPh_3_)_8_]^2+^	OPAUPF^81^
[Au_8_S_2_(dppm)_4_]^2+^	LEVKIJ^27^
[Au_9_(PPh_3_)_8_]^3+^ (*D*_2h_)	MIVPOX^82^
[Au_11_(PMePh_2_)_10_]^3+^ (*C*_3v_)	ZUCMAL^83^
[Au_11_(PMePh_2_)_10_]^3+^ (*D*_4d_)	ZUCMEP^83^
[Au_13_(dppm)_6_]^5+^	LEVKAB^27^
[Au_20_(PP_3_)_4_]^4+^	POFPUX^84^
Au_22_(dppo)_6_	TOCFIC^85^
[Au_38_(*m*-MBT)_20_(PPh_3_)_4_]^2+^	CEMZIG^86^
Au_70_S_20_(PPh_3_)_12_	TELMUV^26^

To test the accuracy of the DFTB parameters for the prediction of vibrational spectroscopic data, we compared DFTB- and PBE-calculated far-IR spectra to the experimental spectra for [Au_6_(dppp)_4_]^2+^, [Au_8_(PPh_3_)_8_]^2+^ and [Au_9_(PPh_3_)_8_]^3+^ clusters. The DFTB2 IR vibrational spectroscopy calculations were computed using the GAMESS-US code.^87,88^ The simulated IR spectra were obtained by convoluting the calculated stick spectra using a Lorentzian line shape function with 3 cm^−1^ full width at half maximum.

The transferability of the DFTB parameters was evaluated by comparing DFT and DFTB energy landscapes of PH_3_ adsorption on the Au(111) surface. A 4 × 4 supercell consisting of 4 layers of Au atoms was cut from bulk and a vacuum layer of 20 Å was added to effectively suppress through-space slab–slab interactions. For the adsorption energy scans, all Au atoms were fixed. Selective geometry relaxation was used by constraining the P atom of the PH_3_ molecule in all but the *z*-direction, while the H atoms the were fully optimized. For the DFT calculations, the PBE functional was used with the projector augmented wave (PAW) approach,^89^ the kinetic energy cutoff was 450 eV, and *k*-point grids were generated dynamically using a 3 × 3 × 1 Monkhorst–Pack scheme. The DFT calculations were carried out with the Vienna *Ab initio* simulation package (VASP) program in conjunction with the provided “PAW_PBE” pseudopotentials.^90,91^ The convergence criteria were set to 10^−6^ eV for achieving self-consistent field energies, and 0.005 eV Å^−1^ for the maximum force in case of geometry optimizations.

For the demonstration application, a case study on the large Au_108_S_24_(PPh_3_)_16_ metalloid was performed. The initial positions of Au, S, and P atoms were taken from the experimental crystal structure (CSD code: DAFLOO). The PH_3_ and PPh_3_ ligands were added based on the position of the P atoms in the CIF file. Then, these complexes went through a two-step geometry optimization: first, only C and H atoms were optimized while Au, S and P atoms were fixed, and second, all atoms were optimized. The fully relaxed complexes were used to calculate binding energy, electronic properties and the IR spectrum.

## 3 Results and discussion

### 3.1 Performance for small- and moderate-sized clusters

#### Small-sized clusters

**Fig. 1:**
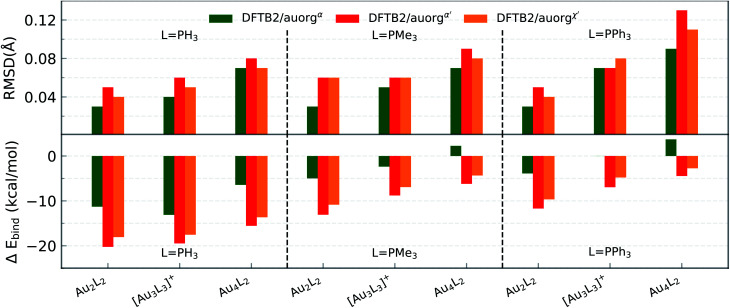
RMSD over atomic positions (upper panel), and deviation in averaged and normalized ligand binding energies (lower panel) for the small-sized gold clusters with different phosphine ligands (L = PH_3_, PMe_3_, and PPh_3_). The RMSD over atomic positions only considers Au and P atoms.

The differences between DFTB and TPSS/def2-SVP levels of theory for optimized geometries and ligand binding energies were evaluated for nine model complexes, which were constructed from three small-sized gold clusters Au_*n*_ (*n* = 2, 3, and 4) with three phosphorus-based donor ligands, including PH_3_, PMe_3_ and PPh_3_. Overall, Fig. 1 shows that all three parameter sets are performing very well, as measured by the root mean square deviation (RMSD) over Au and P atom positions, with all values below 0.13 Å. auorg^*α*^ is superior among the three sets with a maximum RMSD of only 0.09 Å, while auorg^*α*′^ produces typically the largest error. The averaged and normalized DFTB ligand binding energy generally shows overbinding for these small-sized complexes relative to the reference TPSS/def2-TZVP level of theory, strongest for auorg^*α*′^ and least for auorg^*α*^ (see Fig. 1 and Table S2 in the ESI†). We note that the deviation in ligand binding energies decreases with the size of gold clusters as well as the size of the ligands: errors are largest for the PH_3_ ligand and smallest for the PPh_3_ ligand. The variation of the binding energy deviation can be attributed to the electronic interaction rather than the repulsive potential: while all Au–P bond lengths in these complexes are almost the same, ≈2.35 Å, the averaged and normalized ligand binding energies increase with the Au cluster size as well as ligand size. For instance, the ligand binding energies of PH_3_, PMe_3_, PPh_3_ with Au_4_ cluster are −33.91, −46.44, and −48.69 kcal mol^−1^ respectively. Since the overbinding can be caused by low virtual orbital energies, it makes sense that the auorg^*α*′^ is mostly affected by it. As for the ranking in performance for averaged and normalized ligand binding energies, we find the same order as for the geometries: auorg^*α*^ is the best parameter in terms of binding energy, while auorg^*α*′^ performs the worst.

#### Moderate-sized clusters

**Fig. 2:**
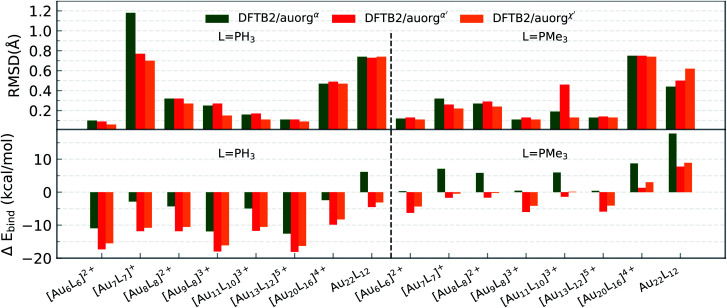
RMSD over atomic positions (upper panel), and deviation in averaged and normalized ligand binding energies (lower panel) for the moderate-sized phosphine-stabilized gold clusters with phosphine ligands (L = PH_3_ and PMe_3_). The RMSD over atom positions only considers Au and P atoms.

Fig. 2 displays the same performance data for moderate-sized clusters Au_*n*_ (*n* = 6–22) with phosphine or trimethylphosphine ligands. The DFTB optimized geometries agree acceptably well with those from TPSS/def2-SVP, with RMSDs less than 0.5 Å in most cases. Exceptionally, three cases of [Au_7_(PH_3_)_7_]^+^, Au_22_(PH_3_)_12_ and [Au_20_(PMe_3_)_16_]^4+^ complexes exhibit RMSD values larger than 0.7 Å. While the RMSD of auorg^*α*′^ and auorg^*χ*′^ for these three clusters, and auorg^*α*^ for Au_22_(PH_3_)_12_ [Au_20_(PMe_3_)_16_]^4+^ remain in an acceptable range around ≈0.7 Å, the auorg^*α*^ RMSD value of 1.18 Å appears problematic for [Au_7_(PH_3_)_7_]^+^.

After closer inspection, we conclude that this large value results from a strongly distorted Au_7_ core shape that can only be stabilized when larger ligands such as PMe_3_ or PPh_3_ are used. It is worth mentioning that these model systems are experimentally not stable in general, because the gold core prefers a planar structure rather than a 3D geometry, unless ligands are present.^92,93^ The model geometries here were constructed by replacing the experimentally used ligands (listed in Table 3) with PH_3_ or PMe_3_ ligands. For these hypothetical models, large RMSDs are expected because the presence of many local minima on the potential energy surfaces complicates the geometry optimization, and can cause the DFT and DFTB geometry optimization to converge to different local minima. For the [Au_7_(PH_3_)_7_]^+^ cluster, lower RMSDs were observed for auorg^*α*′^ and auorg^*χ*′^ because these two parameters increase the contribution of the P 3d-orbital in stabilizing the complex due to its lower orbital energy.

Compared to the situation in small-sized clusters, ligand overbinding is less prominent in the medium-sized systems for both auorg^*α*′^ and auorg^*χ*′^ parameters, with energy deviations as high as −18 kcal mol^−1^ for the smallest PH_3_-ligated clusters (see Fig. 2 and Table S3 in the ESI†). Again we find generally that the averaged and normalized ligand binding energy deviations become smaller when the larger PMe_3_ ligands are used. Again, auorg^*α*^ has smaller overbinding than the other two parameters for PH_3_ ligands, and this tendency turns to underbinding when the larger PMe_3_ ligands are used. The performance ranking for the three parameter sets is less clear-cut as in the case of the small-sized clusters, but especially in terms of ligand binding energies we find the tendency confirmed that auorg^*α*^ tends towards least overbinding and auorg^*α*′^ towards greatest overbinding, with auorg^*χ*′^ somewhere in between but closer to auorg^*α*′^. Since small ligands themselves consistently tend to increase overbinding, we find that auorg^*α*^ performs best for small ligands and small Au clusters, while auorg^*α*′^ or auorg^*χ*′^ parameters are better for larger ligands and larger gold clusters.

### 3.2 Performance for large-sized clusters

The accuracy of the new DFTB2 parameters for geometries and ligand binding energies was further assessed for a series of gold clusters (Au_*n*_, *n* = 6–70) in complexes with their experimentally used larger ligands including PPh3, PMePh_2_, PP_3_, dppp, dppm, dppo, and *m*-MBT, see Table 3. In this test, the experimental crystal structures were used as the reference geometry for RMSD evaluation, rather than DFT geometries. In both DFT and DFTB calculations, all counterions were removed and no symmetry constrains were applied. In this section, only the performance of auorg^*α*^ and auorg^*α*′^ parameters is presented and discussed in the main text. The performance of the auorg^*χ*′^ parameter is presented in the ESI.†

#### Geometry

**Fig. 3:**
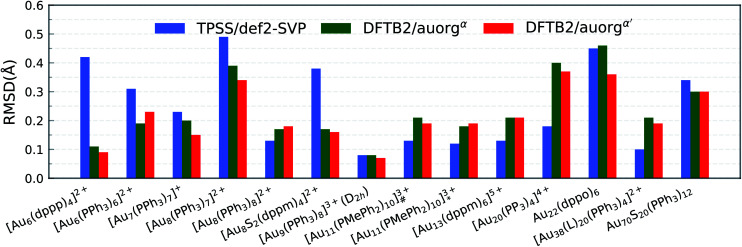
RMSD over atomic positions for the large-sized phosphine-stabilized gold clusters. The RMSD of atomic positions considers Au, and P atoms for all large-sized phosphine-based gold clusters, [Au_11_(PMePh_2_)_10_]^3+^_#_ denotes [Au_11_(PMePh_2_)_10_]^3+^ (*C*_3v_), [Au_11_(PMePh_2_)_10_]^3+^_*_ denotes [Au_11_(PMePh_2_)_10_]^3+^ (*D*_4d_), [Au_38_(L)_20_(PPh_3_)_4_]^2+^ denotes [Au_38_(*m*-MBT)_20_(PPh_3_)_4_]^2+^.

The RMSDs between experimental and computed cluster geometries shown in Fig. 3 demonstrate that the DFTB2 methods are able to reproduce X-ray structures, with maximum RMSDs equal to 0.46 Å and 0.37 Å for auorg^*α*^ and auorg^*α*′^, respectively. The same RMSDs of auorg^*χ*′^ are shown in Fig. S2 in the ESI.† In fact, the DFTB2 geometries even outperform TPSS/def2-SVP geometries for many clusters in the test set, as for instance in the case of [Au_6_(dppp)_4_]^2+^ (BOTSOS), [Au_6_(PPh_3_)_6_]^2+^ (CATPAO10), and [Au_8_S_2_(dppm)_4_]^2+^ (LEVKIJ). In the previous section, [Au_7_(PH_3_)_7_]^+^, Au_22_(PH_3_)_12_ and [Au_20_(PMe_3_)_16_]^4+^ model clusters were found to be the most problematic cases. Here, with the experimentally used ligands, DFTB-optimized structures of [Au_7_(PPh_3_)_7_]^+^, [Au_20_(PP_3_)_4_]^4+^, and Au_22_(dppo)_6_ have lower RMSD values than their previously described truncated model clusters with PH_3_ and PMe_3_. auorg^*α*′^ slightly outperforms auorg^*α*^ in most of the cases (by only up to 0.1 Å for the [Au_22_(dppo)_6_] neutral complex). It is important to keep in mind that the RMSDs over atom position convolutes deviations in angles and torsions with the bond lengths. If one only considers bond length comparison, Au–Au and Au–P deviations are within 0.06 Å from experimental values.

**Fig. 4:**
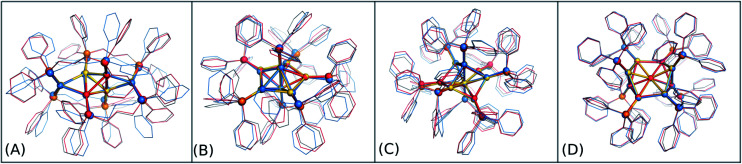
Overlap of experimental crystal structure (Au in gold, P in orange and C in grey) and optimized auorg^*α*′^ and DFT structures. auorg^*α*′^ and DFT structures are represented by light red and sky blue, respectively. The gold nanoclusters considered in this figure are (A) [Au_6_(dppp)_4_]^2+^ (BOTSOS), (B) [Au_7_(PPh_3_)_7_]^+^ (BIXZAK), (C) [Au_8_(PPh_3_)_8_]^2+^ (OPAUPF), and (D) [Au_9_(PPh_3_)_8_]^3+^ (MIVPOX-*D*_2h_).

In order to graphically illustrate the differences between experimental and computed DFT and auorg^*χ*′^ geometries, Fig. 4 shows X-ray and optimized structures for the following ligated Au clusters: [Au_6_(dppp)_4_]^2+^ (BOTSOS), [Au_7_(PPh_3_)_7_]^+^ (BIXZAK), [Au_8_(PPh_3_)_8_]^2+^ (OPAUPF), and [Au_9_(PPh_3_)_8_]^3+^ (MIVPOX-*D*_2h_). The analogous comparison for auorg^*α*^ and auorg^*χ*′^ is shown in Fig. S3 in the ESI.† The overlapped structures were determined by a minimization procedure, which includes recentering and rotation to minimize the RMSD using the quaternion algorithm.^94^ The figure highlights the good performance of the theoretical methods in the description of the cluster core geometries relative to experiment. We note that ligand orientations can be strongly impacted by crystal field effects that are not present in our gas phase theoretical calculations, and thus we will not discuss differences in ligand geometries.

#### Binding energy

**Fig. 5:**
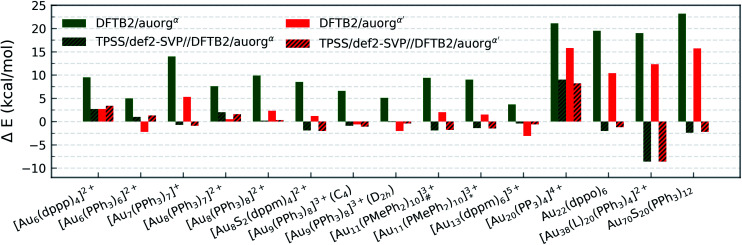
Deviation in averaged and normalized ligand binding energies for the large-sized phosphine-stabilized gold clusters in reference to the TPSS/def2-SVP binding energies, [Au_11_(PMePh_2_)_10_]^3+^_#_ denotes [Au_11_(PMePh_2_)_10_]^3+^ (*C*_3v_), [Au_11_(PMePh_2_)_10_]^3+^_*_ denotes [Au_11_(PMePh_2_)_10_]^3+^ (*D*_4d_), [Au_38_(L)_20_(PPh_3_)_4_]^2+^ denotes [Au_38_(*m*-MBT)_20_(PPh_3_)_4_]^2+^.

The evaluation of predicted averaged and normalized ligand binding energies follows the schemes used above for the smaller-sized and moderate-sized clusters. The ligand binding energies are listed in Table S4 in the ESI,† the relative deviations of the DFTB methods from TPSS/def2-SVP data are shown in Fig. 5 for auorg^*α*^ and auorg^*α*′^, and Fig. S4 in the ESI† for auorg^*χ*′^.

All predictions by DFTB tend towards underbinding, with only three minor exceptions. From the smallest [Au_6_(dppp)_4_]^2+^ up to [Au_13_(dppm)_6_]^5+^, the auorg^*α*′^ binding energy deviations of ≤5 kcal mol^−1^ are in very good agreement with the DFT reference. auorg^*α*^ trends towards strong underbinding in all cases. In the case of clusters with more core Au atoms (*n* ≥ 20), auorg^*α*^ and auorg^*α*′^ have both noticeable underbinding as high as 20 and 15 kcal mol^−1^, respectively. The results are consistent with the overall trend that was already seen in the small-sized and moderate-sized ligand complexes above. To further investigate the effect of geometry on the ligand binding energies, TPSS single point energy calculation using the DFTB optimized geometries were carried out, and the corresponding deviations in ligand binding energies are shown in Fig. 5 and S4 in the ESI,† indicated as usual by the at-the-geometry-of symbol “//” symbol. It becomes immediately obvious that the deviation of ligand binding energies with TPSS single point energy refinement is reduced in all cases, with a maximum absolute deviation of ≤9 kcal mol^−1^. It follows that, if highly accurate ligand binding energies are required, DFTB geometry optimizations followed by TPSS single point energy calculations can provide a reasonable “shortcut” over straightforward DFT calculations. In addition to the binding energies, DFTB isomerization energies are compared to the DFT values as well for [Au_9_(PPh_3_)_8_]^3+^ and [Au_11_(PMePh_2_)_10_]^3+^ clusters, see Fig. S5 in the ESI† for more details.

### 3.3 Gold cluster–ligand bond dissociation energy curves for the [Au_8_(PPh_3_)_8_]^2+^ complex

**Fig. 6:**
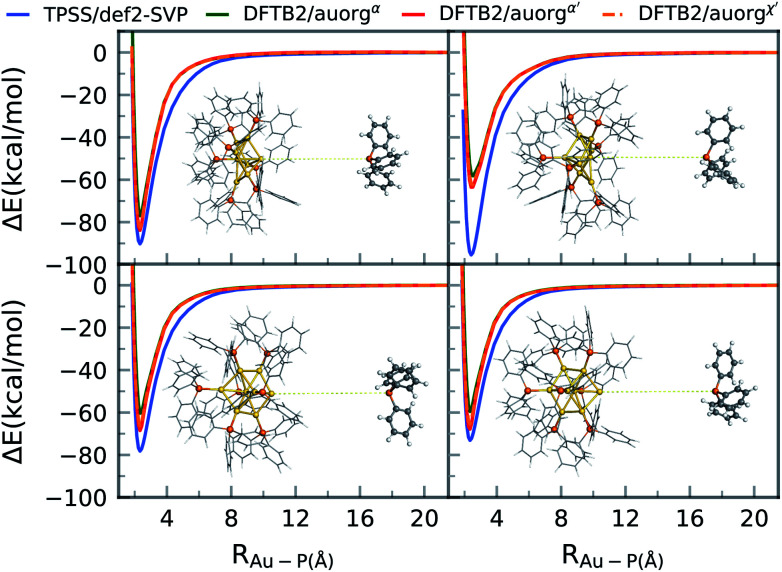
Gold cluster–ligand rigid bond dissociation energy curves of [Au_8_(PPh_3_)_8_]^2+^.

Ligand removal of nanoscale gold clusters is a key step in making the clusters catalytically more active by increasing the gold core interaction with the substrate or reactants. Previous studies have reported the partial removal of ligands of atomically-precise gold clusters Au_*n*_(PPh_3_)_*m*_ (*n* = 8, 9, 11, and 101) on titania after undergoing different treatments such as calcination and acid-washing.^10,11^ One of the primary applications of the DFTB methodology developed here is the theoretical study of cluster fragmentation and the catalytic reaction mechanisms of clusters with dissociated ligands. For validation against DFT, we therefore compare rigid energy scans for Au–P bond dissociation between TPSS/def-SVP and DFTB2 methods for the aforementioned [Au_8_(PPh_3_)_8_]^2+^ cluster as a representative for the experimentally relevant complexes with larger ligands. Starting from the DFT optimized geometries of the cluster, the Au–P bond of the ligand in question was simply stretched up to a distance of 20 Å. The relative energy Δ*E* of this practically dissociated geometry was defined in all methods as 0 kcal mol^−1^. In reality, complex structural relaxation of the cluster and ligand would occur obscuring methodological differences, which is the reason for presenting rigid scans. Fig. 6 shows four different bond dissociation energy curves that correspond to four ligand detachment scans.

Overall, the DFTB curves mimic the TPSS curves closely in both energy of the binding region and the shape of the energy curves. The rigid scan does not include a barrier and converges within about 12 Å to the dissociation limit. As for the DFTB curves, we observe underbinding in the region from 2.5 to 6 Å in all of the plots (*cf.*Fig. 5). DFTB is strongly underbinding in the case of one ligand by more than 30 kcal mol^−1^ (upper right curve in Fig. 6). Here, the Au atom involved in the Au–P bond has a more “surface”-like binding with the other Au atoms as opposed to a pyramidal shape as in the other ligand cases. For more “cluster”-like detached Au atoms, the deviations are in the range of ≈5–10 kcal mol^−1^. In all cases, DFTB2/auorg^*α*′^ and DFTB2/auorg^*χ*′^ are very similar to each other and outperform DFTB2/auorg^*α*^. As is typical for the DFTB method in general, the entrance region of the binding well, here around 6 Å, is underbinding since the atomic orbitals are compressed with fixed electronic confinement radii optimized to describe geometries and binding energies.^95^ The underestimation of phosphine ligands binding to gold “surface” will be discussed further below regarding the adsorption of a PH_3_ molecule on the gold (111) surface.

### 3.4 Electronic structures of phosphine-stabilized gold clusters

**Fig. 7:**
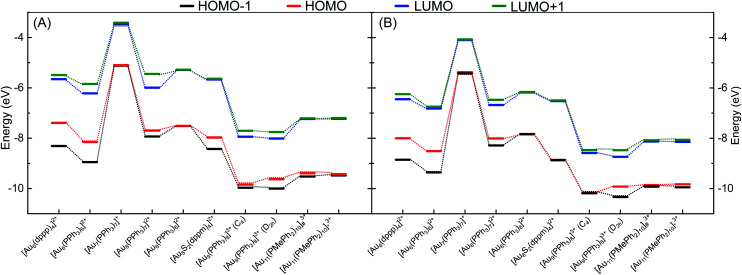
Energy level diagram for the frontier orbitals of various clusters as calculated by (A) TPSS/def2-SVP and (B) DFTB2/auorg^*α*′^, [Au_11_(PMePh_2_)_10_]^3+^_#_ denotes [Au_11_(PMePh_2_)_10_]^3+^ (*C*_3v_), [Au_11_(PMePh_2_)_10_]^3+^_*_ denotes [Au_11_(PMePh_2_)_10_]^3+^ (*D*_4d_). Dashed lines are included to guide the eye.

To evaluate the ability of DFTB to accurately describe electronic structures of phosphine-stabilized gold clusters, we investigated the frontier orbital energies (HOMO-1, HOMO, LUMO, LUMO+1) and HOMO–LUMO gaps (HLGs) of a number of medium-sized gold clusters using DFT and DFTB methods. In this discussion, we concentrate on the auorg^*α*′^ parameter, but show the corresponding performance of the other parameters in the ESI.†Fig. 7 shows the TPSS/def2-SVP and DFTB/auorg^*α*′^ calculated energy levels of the frontier molecular orbitals for selected gold clusters. The plots showing frontier orbitals computed with auorg^*α*′^ and auorg^*χ*′^ are shown in Fig. S5 in the ESI.† The total charge in the selected clusters changes from +1 to +3, with the orbitals of the least charged clusters being highest in absolute orbital energies, and the highest charged clusters having lowest absolute orbital energies. Within the energy range spanned by these four orbitals among these clusters, the DFTB orbital energy levels reproduce TPSS orbital energy levels very well, as indicated by the dashed lines to mark the changes of electronic structure with molecular structure and total charge. Individual orbital energy level shifts are appreciable and may reach ≈0.9 eV (see Table S6†), with a general trend towards lower energies. Regarding HLGs, DFTB/auorg^*α*′^ tends to underestimate those for the singly charged [Au_7_(PPh_3_)_7_]^+^ (BIXZAK), the doubly charged [Au_8_(PPh_3_)_8_]^2+^ (OPAUPF), and the triply charged [Au_11_(PMePh_2_)_10_]^3+^ (ZUCMEP) clusters. In these particular three clusters, the DFTB HOMO energy shifts are −0.28, −0.32, and −0.40 eV for Au_7_, Au_8_ and Au_11_ respectively, while their LUMO shifts are −0.63, −0.89, −0.92 eV, obviously quantitatively larger. This imbalance results in their smaller HOMO–LUMO gap values. Nonetheless, the HLG values of DFTB/auorg^*α*′^ are quantitatively close to the TPSS calculated values with deviations no greater than 0.6 eV.

Fig. S7, in the ESI,† displays the HOMO and LUMO orbital shapes for [Au_6_(dppp)_4_]^2+^, [Au_7_(PPh_3_)_7_]^+^ (BIXZAK), [Au_8_(PPh_3_)_8_]^2+^ (OPAUPF) and [Au_9_(PPh_3_)_8_]^3+^ (MIVPOX-*D*_2h_) clusters for the TPSS and DFTB2/auorg^*α*′^ methods. Despite some inevitable quantitative differences in the orbital topology, the amplitudes of the HOMO and LUMO orbitals obtained with the DFTB2/auorg^*α*′^ method are very close to the ones obtained by TPSS/def2-SVP. Noticeable qualitative differences are the LUMOs of Au_7_ and Au_8_ where DFTB both overestimated the contribution from the gold core and less contributions from the phosphine ligands, possibly providing a rationale for the previously discussed large LUMO energy shifts of Au_7_ and Au_8_. The HOMO and LUMO plots of DFTB2/auorg^*α*^ and DFTB2/auorg^*χ*′^ are shown in Fig. S8 and S9 in the ESI.† Both of these two sets of molecular orbitals have similar shapes with DFTB2/auorg^*α*′^ orbitals; with DFTB2/auorg^*α*^ also overestimating contribution from the gold core while DFTB2/auorg^*χ*′^ underestimates the gold and overestimates the contribution from phosphine ligands in Au_7_. We note that these differences within the different DFTB parameter sets originate mostly from the different choices of the virtual orbital energies (see Table 1) and a minor degree from the different geometries.

### 3.5 IR spectra of [Au_6_(dppp)_4_]^2+^, [Au_8_(PPh_3_)_8_]^2+^ and [Au_9_(PPh_3_)_8_]^3+^ clusters

**Fig. 8:**
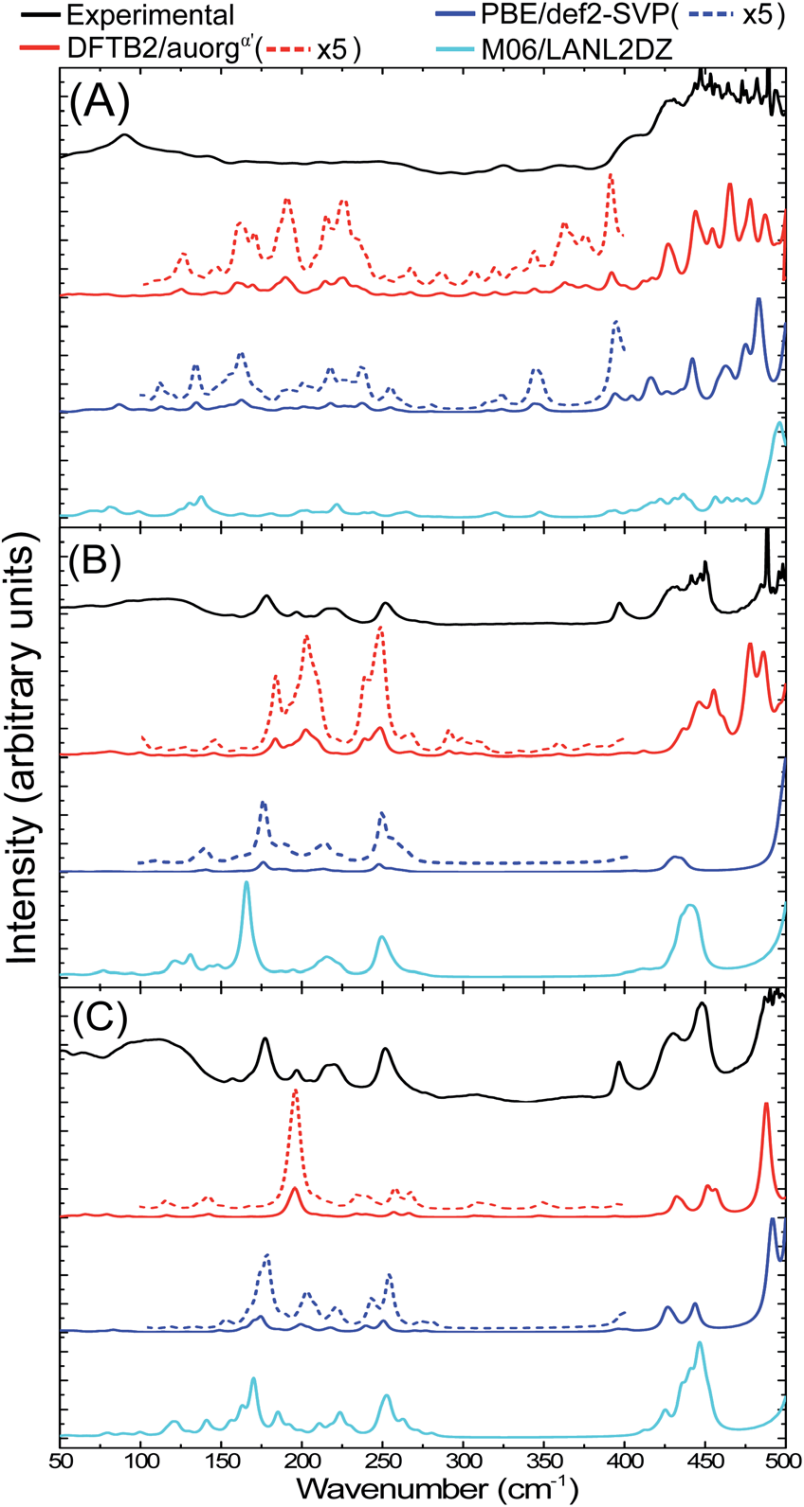
Experimental (in black),^19^ computed DFTB2/auorg^*α*′^ (in red), PBE/def2-SVP (in blue) and M06/LANL2DZ (in cyan) far-IR spectra for (A) [Au_6_(dppp)_4_]^2+^, (B) [Au_8_(PPh_3_)_8_]^2+^, and (C) [Au_9_(PPh_3_)_8_]^3+^ clusters. The additional red and blue dashed lines are for the scaled up plots of the region 100–400 cm^−1^ for DFTB2/auorg^*α*′^ and PBE/def2-SVP.

The IR spectra of [Au_6_(dppp)_4_]^2+^ (BOTSOS), [Au_8_(PPh_3_)_8_]^2+^ (OPAUPF), and [Au_9_(PPh_3_)_8_]^3+^ (MIVPOX-*D*_2h_) clusters have been studied previously both experimentally and computationally using DFT^19^ and therefore represent good choices for the performance of our DFTB parameters for predicting vibrational spectra. The calculated DFTB IR spectra were evaluated by comparing them with experimental data and DFT-calculated IR, see Fig. 8 and S10 in the ESI.† The experimental far-IR spectra of the three clusters consists of two main parts: gold core distortion between the range 90–250 cm^−1^ and Au–P modes above 400 cm^−1^. Both PBE/def2-SVP and DFTB2/auorg^*α*′^-calculated IR spectra match the previously reported experimental and M06/LANL2DZ predicted spectra, having the same mode description for each vibration. Also, the DFTB and PBE normalized spectral shapes are in good agreement, especially concerning the main peaks involving Au–P interactions. A noticeable difference between the experimental and calculated spectra of Au_8_ and Au_9_ is the presence of an experimentally distinct peak at 398 cm^−1^. While all DFT and DFTB methods are able to predict peaks that are comparable with the position of this feature, the calculated intensities in DFTB are considerably weaker. Nevertheless, both methods have shown that this peak can be attributed to phenyl group twisting vibrations. Another difference is the presence of a peak at 317 cm^−1^ in the Au_8_ and Au_9_ DFTB calculated spectra that is missing in the DFT spectra. The DFTB peak matches a seemingly broad experimental peak in the same region. In the previous study,^19^ this peak was not assigned to any vibrational mode, as DFT was not able to predict this feature. DFTB revealed that these peaks near 317 cm^−1^ of Au_8_ are related to vibrations with weaker intensity assigned to a phenyl rocking vibration that is attached to protruding Au_1_–P_8_. In the case of Au_9_, this peak has several contributing vibrations that are also assigned to a phenyl rocking of the complex's several phosphine ligands. The summary of contributing transitions for all clusters are given in Tables S7–S9 in the ESI.† We conclude that DFTB is very useful in the prediction of vibrational spectra for nanoscale gold clusters with stabilizing ligands; this had not been discussed in the previous auorg parameterization work.^58,59^

### 3.6 Chemisorption of PH_3_ on the Au (111) surface

**Fig. 9:**
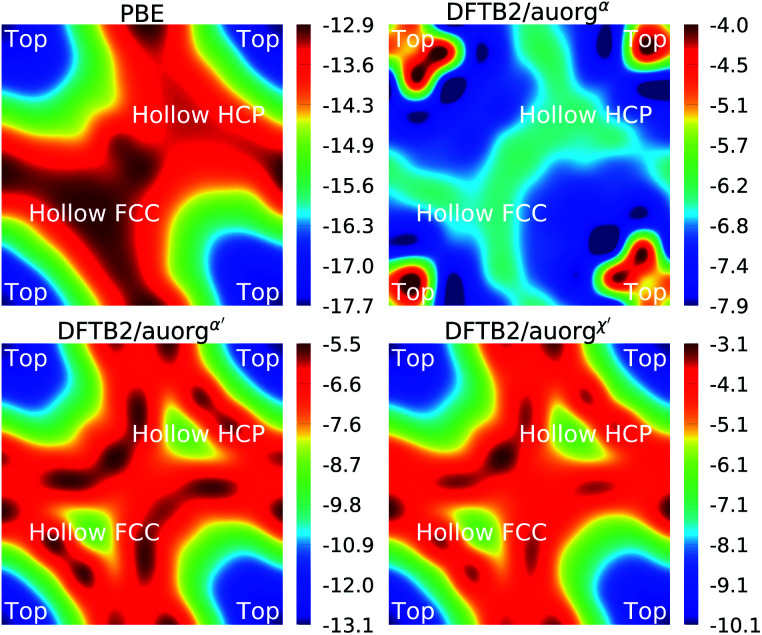
Energy landscape in kcal mol^−1^ of PH_3_ adsorption on the Au (111) surface obtained at the PBE and DFTB2 methods. The energy profiles show the three important adsorption locations of the Au (111) surface for clear comparison.

To evaluate the transferability of the new DFTB parameters, we explored the energy landscape of PH_3_ chemisorption on the Au (111) surface and compared it to DFT with the PBE functional, which is popular in the solid state community to simulate surface processes. The computed DFTB2/auorg^*α*^, DFTB2/auorg^*α*′^, DFTB2/auorg^*χ*′^ and PBE energy landscapes are shown in Fig. 9. In this Au (111) surface model, there are three important adsorption locations: top, hollow fcc, and hollow hcp (see Fig. S11 in the ESI†). The corresponding top and hollow adsorption sites are marked in Fig. 9 for reference. This test is particularly useful for evaluating the transferability of the new parameters, as the number of Au atoms coordinating with the P atom of PH_3_ molecule varies from one at the top site, to two at the bridge sites (see Fig. S11 in the ESI†), to three at the hollow sites. Fig. 9 shows that the adsorption energies vary in a range of approximately 5 kcal mol^−1^, and both auorg^*α*′^ and auorg^*χ*′^ parameters are able to reproduce qualitatively the relative PBE energy landscape. The top site is the most preferred adsorption location of PH_3_ adsorption on the Au (111) surface according to PBE, DFTB2/auorg^*α*′^ and DFTB2/auorg^*χ*′^. However, DFTB2/auorg^*α*^ predicts the most preferred adsorption location is somewhere in the middle of top, hollow fcc, and hollow hcp positions.

Considering the absolute binding energy, all three DFTB parameter sets underestimate the Au–P interaction energy compared to the PBE method. Among them, the underestimation is smallest with the deviation Δ*E* ≈ 4.6–7.4 kcal mol^−1^ for auorg^*α*′^, the deviation Δ*E* ≈ 7.6–9.8 kcal mol^−1^ for auorg^*χ*′^, and largest with Δ*E* ≈ 8.9–9.8 for auorg^*α*^. The trends in the deviations can again be rationalized by correlating them with the orbital energies of the virtual Au 6p and P 3d orbitals: the larger the upward shift, the greater the underbinding. This test shows that DFTB2/auorg^*α*′^ is the best option to study phosphine ligands chemisorbed on Au surfaces, and that our parameter has difficulties for the interaction of phosphines with flat Au surfaces, as mentioned above in Section 3.3.

### 3.7 Case study for Au_108_S_24_(PPh_3_)_16_: geometry, ligand binding energy, frontier orbitals, and IR spectra

**Fig. 10:**
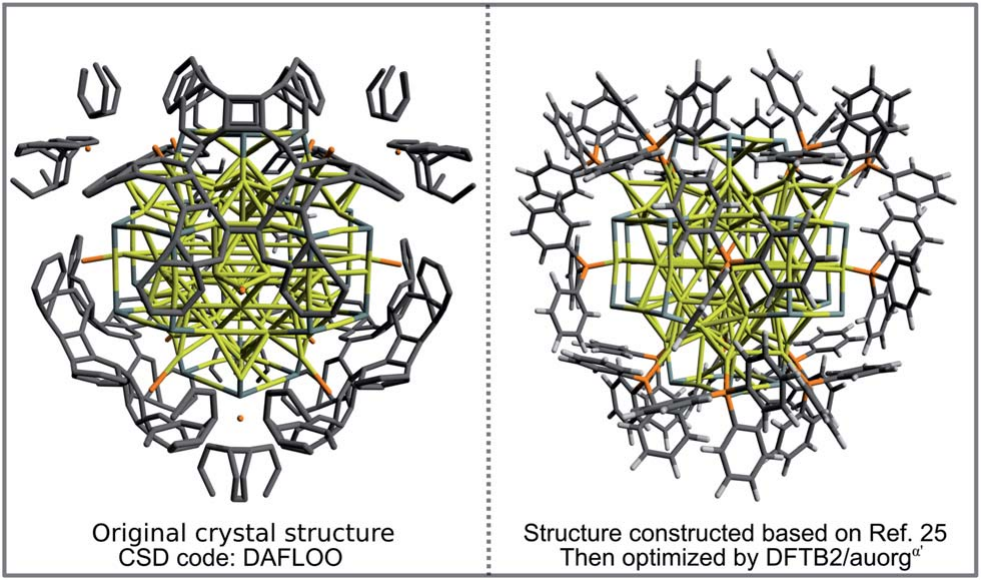
X-ray experimental structure of Au_108_S_24_(PPh_3_)_16_ taken from Cambridge Crystallographic Database (CSD code DAFLOO) with overlapping phenyl rings and ultra short ≈0.2 Å C–C bond lengths between triphenylphosphine groups (left panel), and DFTB2/auorg^*α*′^ optimized structure without overlapping phenyl rings (right panel).

In order to demonstrate the capability of the new DFTB parameters for the study of large-scale phosphine-stabilized gold clusters, we performed a case study for the recently synthesized metalloid complex Au_108_S_24_(PPh_3_)_16_.^25^ The core of the metalloid consists of a Russian doll motif with an Au_6_ inner octahedron enclosed by a second-shell Au_38_ octahedron. This core is then capped at its six tips by novel Au_4_S_4_ planar rings, which are then connected by 4 more gold atoms, resulting in a (Au_2_S)_24_ outer shell motif. Finally, sixteen Au(PPh_3_) groups complete this metalloid and are bound to each side of the Au_44_ octahedral Russian-doll core. The available experimental crystal structure (CSD code: DAFLOO) provides a clear picture of Au, S and P atomic positions in 3D. However, it does not depict the positions of C atoms well due to overlapping rings of the triphenylphosphine groups, with unreasonable C–C distances in the CIF file, sometimes being shorter than 0.2 Å. To prepare the initial geometrical inputs for our DFTB geometry optimization, we constructed the initial structure based on the experimental positions of Au, S and P atoms, and then added manually the sixteen PPh_3_ ligands. The geometry was then optimized using the DFTB2/auorg^*α*′^ method. Fig. 10 compares the final DFTB optimized geometry to the initial, experimental crystal structure. DFTB2/auorg^*α*′^ predicts the averaged and normalized ligand binding energies of Au_108_S_24_(PPh_3_)_16_ to be −63.2 kcal mol^−1^, in good agreement with TPSS/def2-SVP single point energies performed using our DFTB optimized geometries, which predicts the averaged ligand binding energy to be −72.9 kcal mol^−1^. We note that a single point energy + gradient calculation on 16 “Intel Xeon E5-2697 2.30 GHz CPU cores” took 39 935 s (11.09 h) with DFT and only 27 s with DFTB, corresponding to a speed-up factor of nearly 1500×. The DFTB method is therefore the only practical option if one considers routinely carrying out geometry optimizations of large nanoscale gold clusters, or a more challenging molecular dynamics (MD) simulation with a few million steps to reach nanosecond time scales.

Computationally, the electronic structure of this cluster has been approximately investigated previously at the BP86/TZVPP level of theory, using a simplified model in which the triphenylphosphine ligands were replaced by PH_3_ groups, and the entire structure assumed to take *T*_d_ point group symmetry.

**Fig. 11:**
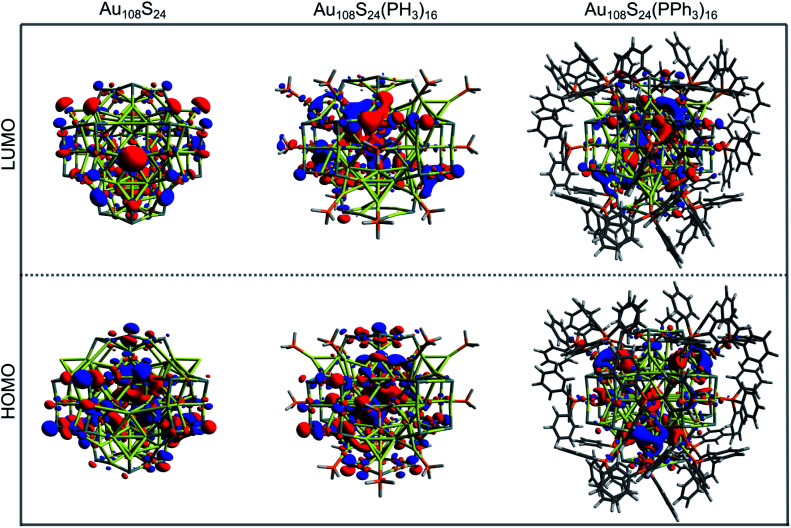
HOMO and LUMO plots of Au_108_S_24_, Au_108_S_24_ (PH_3_)_16_, and Au_108_S_24_(PPh_3_)_16_ clusters as calculated by DFTB2/auorg^*α*′^; isosurface value = 0.015 a.u.

**Fig. 12:**
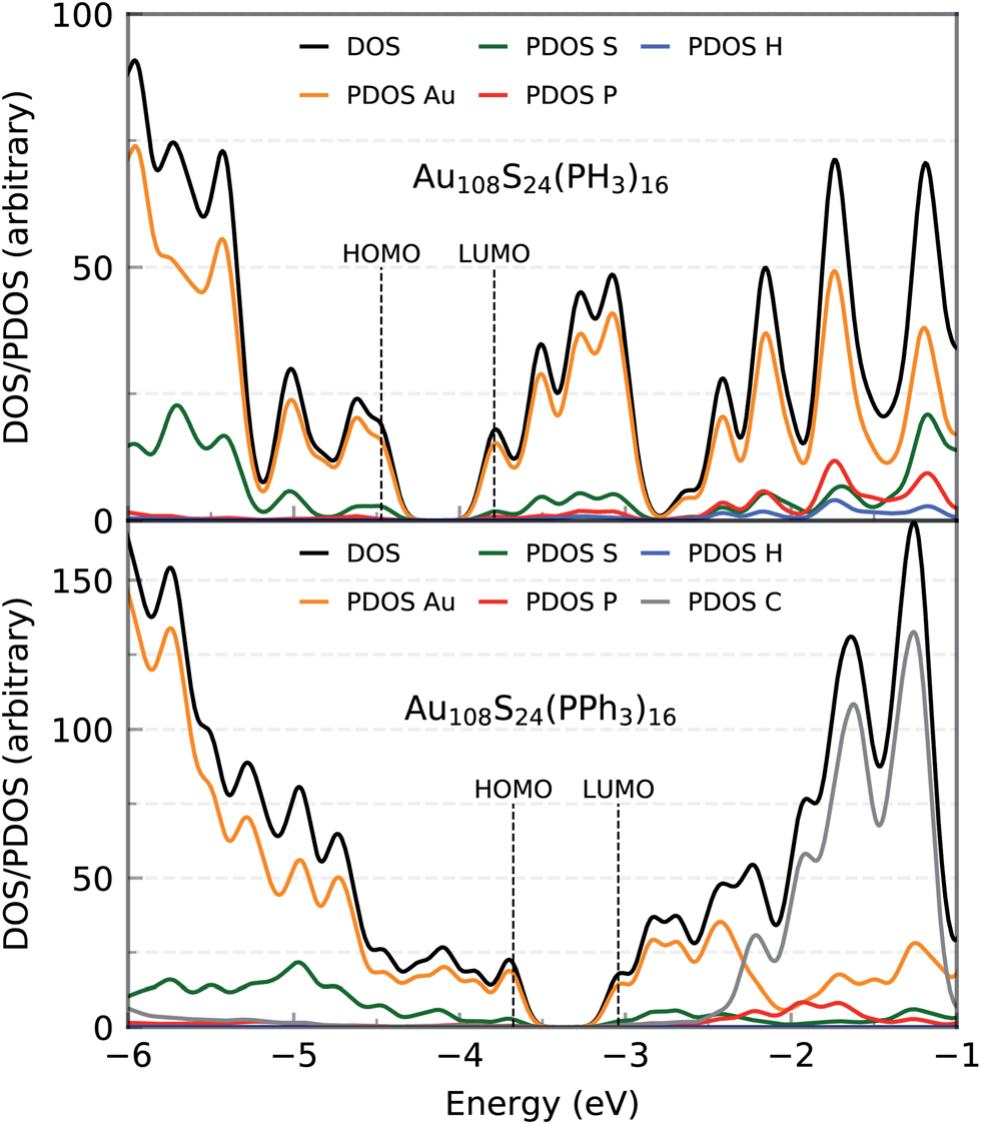
Density of states (DOS) and partial density of states (PDOS) of Au_108_S_24_(PH_3_)_16_ (top) and Au_108_S_24_(PPh_3_)_16_ (bottom) clusters as calculated by DFTB2/auorg^*α*′^.

The HOMO–LUMO gap of this simplified Au_108_S_24_(PH_3_)_16_ cluster was found to be 0.68 eV. Fig. 11 shows the DFTB2/auorg^*α*′^ HOMO and LUMO plots of Au_108_S_24_, Au_108_S_24_(PH_3_)_16_, and Au_108_S_24_(PPh_3_)_16_ clusters. The calculated HOMO–LUMO gaps for these three clusters are 0.200 eV, 0.681 eV, and 0.634 eV, respectively. The increase in the HLG from the Au_108_S_24_ core to fully ligated cluster denotes that the phosphine groups stabilize the core, much like in Au_70_S_20_(PPh_3_)_12_.^26^ The quantitative difference in the energy gaps between Au_108_S_24_(PH_3_)_16_ and Au_108_S_24_(PPh_3_)_16_ clusters are smaller than 0.05 eV. However, it is worth noting that partial density of state (PDOS) plots, see Fig. 12, portray that there is an undeniable difference of sulfur-orbital contribution in the bonding of the Au_108_S_24_(PH_3_)_16_ and Au_108_S_24_(PPh_3_)_16_ clusters. In addition, both HOMO and LUMO are shifted by ≈−0.80 eV when PPh_3_ ligands are replaced by PH_3_ ligands. In the case of the HLG, employing the simplified model Au_108_S_24_(PH_3_)_16_ yields a similar value to the HLG of the Au_108_S_24_(PPh_3_)_16_ cluster, however, the Fermi level has shifted dramatically by −0.8 eV! Our calculations therefore indicate that the use of a simplified model is inadequate and can indeed significantly affect the electronic properties of nanoclusters.

**Fig. 13:**
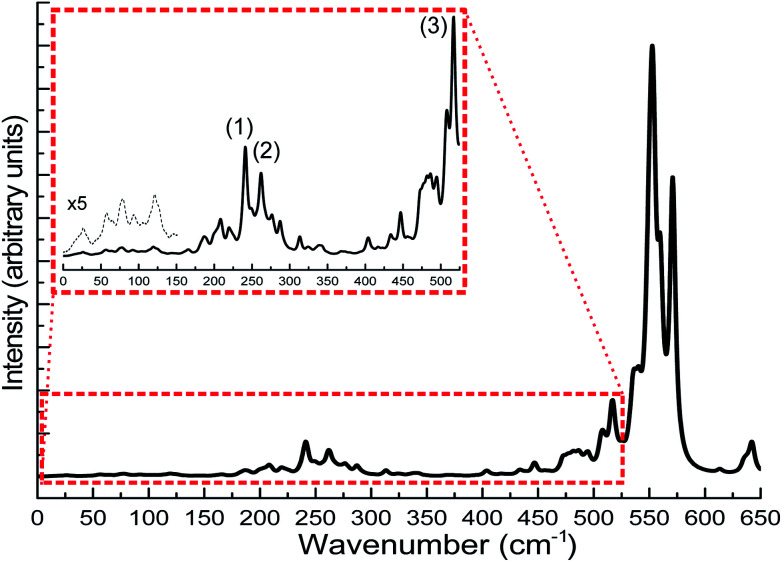
Predicted far-IR spectra for Au_108_S_24_(PPh_3_)_16_ clusters calculated using DFTB2/auorg^*α*′^. The inset of the figure shows the scaled up plots of the region 0–550 cm^−1^ with labelled peaks pertaining to normal modes of Au_4_S_4_ planar rings. The additional black dashed line in the inset is for the scaled-up plot of the region 0–150 cm^−1^.

The calculation of IR spectra of large-scale systems within the normal mode approximation, requiring the calculation of the Hessian matrix, is beyond the capability of most contemporary DFT codes on current computer systems within a reasonable amount of time. In this case, DFTB can be particularly useful as it has been shown in the benchmark sections that DFTB can reproduce the experimental IR spectra very well. In this case study, we carried out DFTB2/auorg^*α*′^ Hessian calculations to predict the IR spectrum for the Au_108_S_24_(PPh_3_)_16_ cluster. Since the performance of DFTB in simulating infrared spectra of thiolate-ligated gold nanoclusters has not been tested before, in this work, we validated the accuracy of DFTB in predicting IR spectra for various thiolated gold clusters. We compare the DFTB simulated IR spectra of Au_4_(SCH_3_)_4_, [Au_25_(SCH_3_)_18_]^−^ clusters, and six Au_18_ clusters protected with various types of ligands to the corresponding DFT and experimental spectra in the case of Au_18_(S-c-C_6_H_11_)_14_.^23,31,32^ The comparison is presented in the ESI, Fig. S13 and S14.† In summary, similar to phosphine-stabilized nanoclusters in Section 3.5, the DFTB-calculated IR spectra for thiolate-protected gold clusters agree well with previous DFT simulations and experimental IR spectra. These new results, in combination with the results presented in Section 3.5, indicate that DFTB is a reliable method in predicting IR spectra of thiolate- and phosphine-stabilized gold nanoclusters. Fig. 13 shows the DFTB calculated far-IR spectrum of Au_108_S_24_(PPh_3_)_16_. We focus our attention on the far-IR region because it is of particular interest in understanding the core vibrations of Au, S, and P atoms. The calculated IR spectrum has several large peaks in the 500–600 cm^−1^ region which are attributed to PPh_3_ distortions. The multiple peaks in the 200–350 cm^−1^ region are caused by various normal modes of both Au–P and Au–S vibrations (see Tables S10–S12 in the ESI†), the peaks labelled in the figure are the normal modes related to the novel Au_4_S_4_ planar rings: (1) Au–S stretching at 241.1 cm^−1^, (2) S–Au–S–Au–S symmetric stretching at 277.2 cm^−1^ and (3) S–Au–S symmetric stretch at 517.05 cm^−1^. The inset of Fig. 13 shows several peaks <150 cm^−1^ which are mostly attributed to Au_44_ core distortion.

Since the novel Au_4_S_4_ planar ring motif of Au_108_S_24_(PPh_3_)_16_ has not yet been studied using IR spectroscopy, there are no experimental validations so far about its normal modes. The calculated Au–S stretches found in this planar ring motif are compared with that of the Au_4_(SCH_3_)_4_, [Au_25_(SCH_3_)_4_]^−^ and [Au_37_(PPh_3_)_10_(SR)_10_Cl_2_]^+^ clusters.^31–33^ The S–Au–S–Au–S asymmetric and symmetric stretching values are found to be 274 cm^−1^ and 277 cm^−1^ (see Peak 2 of the inset in Fig. 13). The S–Au–S–Au–S symmetric stretching value is similar to the DFT-calculated breathing mode (293 cm^−1^) of Au_4_(SCH_3_)_4_ cluster. Other IR studies of thiolate-containing Au nanoclusters such as [Au_25_(SCH_3_)_18_]^−^ and [Au_37_(PPh_3_)_10_(SR)_10_Cl_2_]^+^ have reported their Au–S symmetric stretches at 293 cm^−1^ and 239 cm^−1^, respectively, which are relatively close to our DFTB-calculated Au–S stretch values; 241 cm^−1^ as shown as Peak 1 in the inset of Fig. 13, and 287 cm^−1^ as presented in Tables S11 and S12,† for the Au_108_S_24_(PPh_3_)_16_ cluster. Peak 3 in the inset of Fig. 13 shows another peak at 514 cm^−1^, which is another characteristic S–Au–S symmetric stretch. One can depict the similarity of the Au–S stretches in the 200–300 cm^−1^ regions with that of other sulfur-capped Au clusters.^20,23,31–33,96,97^ Based on the good agreement between our DFTB calculated IR spectra with DFT calculated as well as the experimental spectra for gold clusters in the benchmark, the predicted IR spectrum of Au_108_S_24_(PPh_3_)_16_ is reliable and provides a useful fingerprint to identify this cluster in future studies.

## 4 Conclusions

Parameters for the density-functional tight-binding (DFTB) method were generated to describe gold–phosphorus interactions for simulations of phosphine-stabilized nanoscale gold clusters. We build on preceding works reporting second-order DFTB (DFTB2) parameters for hybrid gold–thiolates compounds,^58,59^ which were themselves based on the mio parameter set.^51,52,57^ The present effort thus expands the applicability of the DFTB2 method to organometallic gold complexes with ligands containing the full set of chemical elements contained in the mio parameter set: H, C, N, O, P, and S. In the construction of the repulsive potentials we considered three different combinations of the gold 6p and phosphorus 3d virtual atomic orbital energies. The performance of our parameters was evaluated using density functional theory (DFT) geometries, ligand binding and cluster isomerization energies, ligand dissociation potential energy curves, molecular orbital energies, and simulated far-IR spectra. We further compare predicted geometries with X-ray crystallographic structures and experimental far-IR spectra, and evaluate parameter transferability for phosphine chemisorption on a gold surface.

In general it is found that the absolute ligand binding energies increase with decreased virtual orbital energies, *i.e.* auorg^*α*^ < auorg^*χ*′^ ≈ auorg^*α*′^. The total ligand binding energy increases both with cluster and ligand size. This means that for a given gold cluster, the variation in the ligand binding energy in DFTB needs to be almost completely described by the electronic energy, as the Au–P bond distances only marginally change with different ligands. However, the current DFTB electronic parameters are not flexible enough to accurately describe the variations in the electronic ligand binding energies. Similarly, the same trend holds for increasing gold cluster size. According to the benchmark results, we found that DFTB2/auorg^*α*^ is the best option to study small ligands and small Au clusters. DFTB2/auorg^*α*′^ is the best option to study large ligands, large Au clusters, or Au surfaces. For the moderate-sized ligands or gold clusters, both DFTB2/auorg^*α*^ and DFTB2/auorg^*α*′^ are good options to be used. The performance of auorg^*χ*′^ for Au–P interactions is similar to that of auorg^*α*′^ because the effects of shifting the Au 6p orbital energy from −0.02786 to −0.00001 hartree is less significant than shifting the P 3d orbital energy from 0.12044 to 0.52044 hartree. However, the effect of shifting the Au 6p orbital energy has not been investigated for Au–(C, H, N, O and S) interactions, thus the use of auorg^*α*′^ is more preferable over auorg^*χ*′^. Besides consideration of cluster and ligand sizes, the geometrical environment of the Au–P bond also influences the performance of our parameters. Surface-like Au atoms present a challenge while more pyramidalized Au atoms are better described. Possible reasons are the minimum basis set or the missing multipolar charge contribution. Such situations are rare, however, and our auorg^*α*′^ parameter performs overall well in theoretical studies of relevant nanoscale gold clusters that experimentally feature large capping ligands. In particular, our DFTB parameterization enables the simulation of ligand dissociation, reliably predicting how post-treatment can be done in experiment without inducing concomitant side-effects such as agglomeration. The auorg^*α*′^ parameter set will be publicly distributed *via* the http://www.dftb.org website. To switch from DFTB2/auorg^*α*′^ to DFTB2/auorg^*α*^, one can simply modify the P 3d-orbital energy according to Table 1.

We employed the new DFTB parameters to determine the geometric structure of an Au_108_S_24_(PPh_3_)_16_ nanocluster^25^ and investigate its molecular and electronic structure. The optimized Cartesian coordinates of Au_108_S_24_(PPh_3_)_16_ are provided in the ESI.† We found that for large ligand-protected gold clusters, using a simplified model in simulations is inadequate and can substantially affect the bonding and electronic structures of the clusters. Finally, we predicted the IR spectrum of the cluster. Both optimized geometric structure and the simulated IR spectrum of the Au_108_S_24_(PPh_3_)_16_ nanocluster will serve as useful reference for future studies.

DFTB approaches with appropriately developed parameters for the description of molecular and electronic structure and energetics as well as vibrational spectra are promising to provide insight as to how to tune catalyst reactivity for both product selectivity and reaction specificity. Further development of DFTB parameters for the interaction of gold clusters with various substrate surfaces will advance the development of transformative catalytic systems. This work is vital to herald the promised potential of unprecedented reaction tunability using cluster-based catalysts.

## Notice of copyright

This manuscript has been authored by UT-Battelle, LLC under Contract No. DE-AC05-00OR22725 with the U.S. Department of Energy. The United States Government retains and the publisher, by accepting the article for publication, acknowledges that the United States Government retains a non-exclusive, paid-up, irrevocable, worldwide license to publish or reproduce the published form of this manuscript, or allow others to do so, for United States Government purposes. The Department of Energy will provide public access to these results of federally sponsored research in accordance with the DOE Public Access Plan (http://energy.gov/downloads/doe-public-access-plan).

## Conflicts of interest

There are no conflicts to declare.

## Supplementary Material

SC-011-D0SC04514D-s001

SC-011-D0SC04514D-s002

SC-011-D0SC04514D-s003

SC-011-D0SC04514D-s004

SC-011-D0SC04514D-s005
