# Supplementary material for: Density-functional tight-binding for phosphine-stabilized nanoscale gold clusters
Source: Chem Sci. 2020 Nov 2;11(48):13113–28. doi: 10.1039/d0sc04514d (PMC8163209; doi:10.1039/d0sc04514d)
Supplement: SC-011-D0SC04514D-s005 [file SC-011-D0SC04514D-s005.pdf]

Electronic Supplementary Material (ESI) for Chemical Science.
This journal is © The Royal Society of Chemistry 2020

Electronic Supplementary Information: Density-Functional Tight-Binding for Phosphine-Stabilized Nanoscale Gold Clusters

Van Quan Vuong,[a‡] Jenica Marie L. Madridejos,[b‡] Bálint Aradi,[c] Bobby G. Sumpter,[de] Gregory F. Metha,[*b] and Stephan Irle[*ae]

List of Figures

S1 The DFTB2 auorg$^{\alpha\prime}$ and auorg$^{\chi\prime}$ Au-P repulsive potentials. Ideally, the DFTB repulsive potentials are positive, however, in the case if Au-P potential the repulsive potential is negative in the bonding range of ≈2.4 Å. The attractive interaction was made to compensate the under-binding of Au-P electronic interaction, which ca be attributed to the effect of the minimum basis set in DFTB method. It is worth mentioning that Au-Au repulsive potential is also attractive ≈16 kcal/mol.[1] . . . 3

S2 RMSD over atomic positions for the large-sized phosphine-stabilized gold clusters. The RMSD of atomic positions considers Au, and P atoms for all large-sized phosphine-based gold clusters, $[Au_{11}(PMePh_2)_{10}]^{3+}_{\#}$ denotes $[Au_{11}(PMePh_2)_{10}]^{3+}$ (C_{3v}), $[Au_{11}(PMePh_2)_{10}]^{3+}_{*}$ denotes $[Au_{11}(PMePh_2)_{10}]^{3+}$ (D_{4d}), $[Au_{38}(L)_{20}(PPh_3)_4]^{2+}$ denotes $[Au_{38}(m{-}MBT)_{20}(PPh_3)_4]^{2+}$. . . . 6

S3 Overlap of experimental crystal structure (Au in gold, P in orange and C in grey) and optimized DFTB/auorg$^{\alpha}$ and DFTB/auorg$^{\chi\prime}$ structures. auorg$^{\alpha}$ and auorg$^{\chi\prime}$ structures are represented by light red and sky blue, respectively. The gold nanoclusters considered in this figure are (A) $[Au_6(dppp)_4]^{2+}$ (BOTSOS), (B) $[Au_7(PPh_3)_7]^{+}$ (BIXZAK), (C) $[Au_8(PPh_3)_8]^{2+}$ (OPAUPF), and (D) $[Au_9(PPh_3)_8]^{3+}$ (MIVPOX-D_{2h}). . . . 6

S4 Deviation in averaged and normalized ligand binding energies for the large-sized phosphine-stabilized gold clusters in reference to the TPSS/def2-SVP binding energies, $[Au_{11}(PMePh_2)_{10}]^{3+}_{\#}$ denotes $[Au_{11}(PMePh_2)_{10}]^{3+}$ (C_{3v}), $[Au_{11}(PMePh_2)_{10}]^{3+}_{*}$ denotes $[Au_{11}(PMePh_2)_{10}]^{3+}$ (D_{4d}), $[Au_{38}(L)_{20}(PPh_3)_4]^{2+}$ denotes $[Au_{38}(m{-}MBT)_{20}(PPh_3)_4]^{2+}$.

S5 Overlap of experimental X-ray (Au in gold, P in orange and C in grey) and optimized structures of (A) $[Au_9(PPh_3)_8]^{3+}$ D_{2h} and C_4 isomers and (B) $[Au_{11}(PMePh_2)_{10}]^{3+}$ D_{4d} and C_{3v} isomers. auorg$^{\alpha\prime}$ and DFT structures are represented by light red and sky blue, respectively. The relative energies in kcal/mol with respect to the D_n isomers are also shown as calculated by TPSS/def-SVP, DFTB2/auorg$^{\alpha\prime}$ and TPSS/def2-SVP//DFTB2/auorg$^{\alpha\prime}$. Note that the $[Au_9(PPh_3)_8]^{3+}$ C_4 isomer does not have an available experimental crystal structure. . . . 7

S6 Energy level diagram for the frontier orbitals of various clusters as calculated by (A) DFTB/auorg$^{\alpha}$ and (B) DFTB2/auorg$^{\chi\prime}$, $[Au_{11}(PMePh_2)_{10}]^{3+}_{\#}$ denotes $[Au_{11}(PMePh_2)_{10}]^{3+}$ (C_{3v}), $[Au_{11}(PMePh_2)_{10}]^{3+}_{*}$ denotes $[Au_{11}(PMePh_2)_{10}]^{3+}$ (D_{4d}). Dashed lines are included to guide the eye. . . . 8

S7 HOMO and LUMO of (A) $[Au_6(dppp)_4]^{2+}$, (B) $[Au_7(PPh_3)_7]^{+}$(BIXZAK), (C)$[Au_8(PPh_3)_8]^{2+}$ (OPAUPF) and (D) $[Au_9(PPh_3)_8]^{3+}$(MIVPOX-D_{2h}) clusters as calculated by TPSS/def2-SVP and DFTB2/auorg$^{\alpha\prime}$; isosurface value = 0.02 a.u. . . . 10

S8 HOMO and LUMO of (A) $[Au_6(dppp)_4]^{2+}$ and (B) $[Au_8(PPh_3)_8]^{2+}$ (OPAUPF) clusters as calculated by TPSS/def2-SVP, DFTB2/auorg$^{\alpha\prime}$, DFTB2/auorg$^{\alpha}$, and DFTB2/auorg$^{\chi\prime}$; isosurface value = 0.02 a.u. . . . 11

S9 HOMO and LUMO of (A) $[Au_7(PPh_3)_7]^{+}$(BIXZAK) and (B) $[Au_9(PPh_3)_8]^{3+}$(MIVPOX-D_{2h}) clusters as calculated by TPSS/def2-SVP, DFTB2/auorg$^{\alpha\prime}$, DFTB2/auorg$^{\alpha}$, and DFTB2/auorg$^{\chi\prime}$; isosurface value = 0.02 a.u. . . . 12

S10 Experimental (in black), computed auorg$^{\alpha}$ (in green) DFTB2/auorg$^{\alpha\prime}$ (in red), and auorg$^{\chi\prime}$ (in orange) far-IR spectra for (A) $[Au_6(dppp)_4]^{2+}$, (B) $[Au_8(PPh_3)_8]^{2+}$, and (C) $[Au_9(PPh_3)_8]^{3+}$ clusters. . . . 12

S11 Different adsorption binding sites on Au (111) surface, only the Au atoms of the top two layers are shown. 16

[a] *Bredesen Center for Interdisciplinary Research and Graduate Education, University of Tennessee, Knoxville, TN, United States*
[b] *Department of Chemistry, The University of Adelaide, South Australia 5005, Australia; E-mail: greg.metha@adelaide.edu.au*
[c] *Bremen Center for Computational Materials Science, University of Bremen, Bremen, Germany*
[d] *Center for Nanophase Materials Sciences, Oak Ridge National Laboratory, Oak Ridge, TN, United States*
[e] *Computational Sciences and Engineering Division, Oak Ridge National Laboratory, Oak Ridge, TN, United States; E-mail: irles@ornl.gov*
‡ These authors contributed equally to this work

S12 HOMO and LUMO plots of $Au_{108}S_{24}$, $Au_{108}S_{24}(PH_3)_{16}$, and $Au_{108}S_{24}(PPh_3)_{16}$ clusters as calculated by TPSS/def2-SVP//DFTB2/auorg$^{\alpha\prime}$; isosurface value = 0.015 $e^{0.5/a_0^3}$. 16
S13 DFTB calculated IR spectra of $Au_4(SCH_3)_4$, $Au_{18}(SCH_3)_{14}$, and $[Au_{25}(SCH_3)_{18}]^-$. 20
S14 Calculated IR spectra of $Au_{18}(SCH_3)_{14}$, $Au_{18}(S-c-C_6H_{11})_{14}$, $Au_{18}(SPh)_{14}$, $Au_{18}(p-MBA)_{14}$, $Au_{18}(SPhNO_2)_{14}$, and $Au_{18}(TBBT)_{14}$ clusters using a FWHM of 5 cm^{-1} Gaussian broadening. More intense IR spectra are observed on p−MBA- and $SPhNO_2$-protected clusters. The additional plots in $S-c-C_6H_{11}$-ligated Au_{18} are the experimental far-IR spectrum (dotted red) and the DFTB-simulated with a wider FWHM of 8 cm^{-1} Gaussian broadening. 20
S15 $Au_{18}S_{14}$ core structures of $Au_{18}(S-c-C_6H_{11})_{14}$, $Au_{18}(p-MBA)_{14}$, and $Au_{18}(SPhNO_2)_{14}$ clusters with four different ligands (ligand structures are omitted for clarity). Au and S atoms are yellow and orange. The clusters are optimized by means of DFTB/auorg$^{\alpha\prime}$ with the D3 dispersion corrections. 21

List of Tables

S1 Test results for selected chemical reactions involving H, C, N, O, P, and S containing compounds for DFTB2/mio with various values of the P 3d orbital energy. The DFTB chemical reaction energies are compared to that of wB97X-D3BJ/def2-TZVP method.[2] The chemical reaction energies and deviations are in kcal/mol, P 3d orbital energies are in Hartree. 4
S2 Averaged and normalized ligand binding ernergies in kcal/mol for small-sized clusters. 5
S3 Averaged and normalized ligand binding ernergies in kcal/mol for moderate-sized clusters. 5
S4 Averaged and normalized ligand binding ernergies in kcal/mol for large-sized clusters, TPSS denotes TPSS/def2-SVP, α denotes DFTB2/auorg$^{\alpha}$, TPSS//α denotes TPSS/def2-SVP//DFTB2/auorg$^{\alpha}$, $\alpha\prime$ denotes DFTB2/auorg$^{\alpha\prime}$, TPSS//$\alpha\prime$ denotes TPSS/def2-SVP//DFTB2/auorg$^{\alpha\prime}$, $\chi\prime$ denotes DFTB2/auorg$^{\chi\prime}$, TPSS//$\chi\prime$ denotes TPSS/def2-SVP//DFTB2/auorg$^{\chi\prime}$. 5
S5 Comparison of relative energies with respect to the D_n isomers in kcal/mol as calculated by the following methods for $[Au_9(PPh_3)_8]^{3+}$ and $[Au_{11}(PMePh_2)_{10}]^{3+}$. 7
S6 Comparison of HOMO and LUMO in eV as calculated by DFTB/auorg$^{\alpha\prime}$, DFTB/auorg$^{\alpha}$, DFTB/auorg$^{\chi\prime}$, and DFT for small gold clusters. 9
S7 Summary of contributing transitions as calculated by PBE/def2-SVP, DFTB2/auorg$^{\alpha\prime}$, DFTB2/auorg$^{\alpha}$ and DFTB2/auorg$^{\chi\prime}$ for the $[Au_6(dppp)_4]^{2+}$ cluster and their assignment to the experimental spectral peaks, as well as a brief description of the assigned transition modes 13
S8 Summary of contributing transitions as calculated by PBE/def2-SVP, DFTB2/auorg$^{\alpha\prime}$, DFTB2/auorg$^{\alpha}$ and DFTB2/auorg$^{\chi\prime}$ for the $[Au_8(PPh_3)_8]^{2+}$ cluster and their assignment to the experimental spectral peaks, as well as a brief description of the assigned transition modes 14
S9 Summary of contributing transitions as calculated by PBE/def2-SVP, DFTB2/auorg$^{\alpha\prime}$, DFTB2/auorg$^{\alpha}$ and DFTB2/auorg$^{\chi\prime}$ for the $[Au_9(PPh_3)_8]^{3+}$ cluster and their assignment to the experimental spectral peaks, as well as a brief description of the assigned transition modes 15
S10 Summary of contributing transitions determined by DFTB2/auorg$^{\alpha\prime}$ for the $[Au_{108}S_{24}(PPh_3)_{16}]$ cluster and brief description of the assigned transition modes, part 1. 17
S11 Summary of contributing transitions determined by DFTB2/auorg$^{\alpha\prime}$ for the $[Au_{108}S_{24}(PPh_3)_{16}]$ cluster and brief description of the assigned transition modes, part 2. 18
S12 Summary of contributing transitions determined by DFTB2/auorg$^{\alpha\prime}$ for the $[Au_{108}S_{24}(PPh_3)_{16}]$ cluster and brief description of the assigned transition modes, part 3. 19

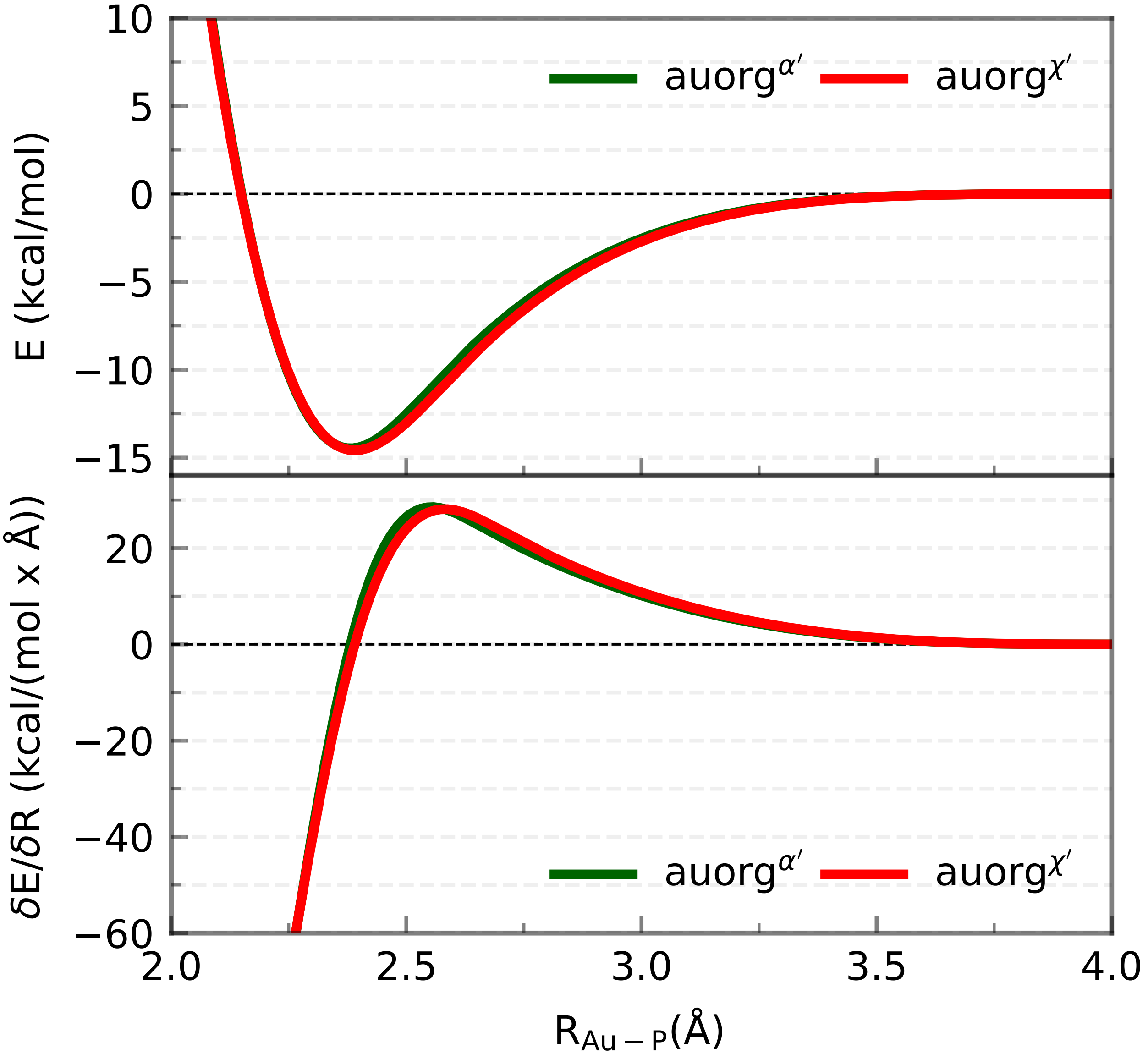

Fig. S1 The DFTB2 auorg$^{\alpha'}$ and auorg$^{\chi'}$ Au-P repulsive potentials. Ideally, the DFTB repulsive potentials are positive, however, in the case if Au-P potential the repulsive potential is negative in the bonding range of ≈2.4 Å. The attractive interaction was made to compensate the under-binding of Au-P electronic interaction, which ca be attributed to the effect of the minimum basis set in DFTB method. It is worth mentioning that Au-Au repulsive potential is also attractive ≈16 kcal/mol.[1]

Table S1 Test results for selected chemical reactions involving H, C, N, O, P, and S containing compounds for DFTB2/mio with various values of the P 3d orbital energy. The DFTB chemical reaction energies are compared to that of wB97X-D3BJ/def2-TZVP method.[2] The chemical reaction energies and deviations are in kcal/mol, P 3d orbital energies are in Hartree.

Reactions					$\Delta E^{react.}$	DFTB2 Deivations						
					wB97XD3BJ	ε_P^{3d}=0.02	ε_P^{3d}=0.07	ε_P^{3d}=0.12	ε_P^{3d}=0.22	ε_P^{3d}=0.32	ε_P^{3d}=0.42	ε_P^{3d}=0.52
P_2	+ P_2	= P_4		⇒	-77.34	23.36	27.24	29.78	32.86	34.63	35.78	36.58
P_2	+ 2 H_2	= H_2PPH_2		⇒	-47.03	39.31	40.00	40.34	40.56	40.55	40.46	40.35
P_2	+ 2 H_3CCH_3	= $H_6C_2PPC_2H_6$		⇒	-40.35	4.71	6.86	8.34	10.27	11.48	12.32	12.93
P_2	+ 3 H_2	= 2 PH_3		⇒	-49.43	65.97	64.82	63.89	62.46	61.42	60.63	60.00
H_2PCH_3	+ H_2	= PH_3	+ CH_4	⇒	-12.93	10.04	9.72	9.48	9.10	8.84	8.64	8.48
$HP(CH_3)_2$	+ 2 H_2	= PH_3	+ 2 CH_4	⇒	-23.99	20.34	19.55	18.94	18.02	17.38	16.90	16.53
$P(CH_3)_3$	+ 3 H_2	= PH_3	+ 3 CH_4	⇒	-33.29	30.81	29.42	28.33	26.74	25.63	24.82	24.19
$OP(CH_3)_3$	+ 3 H_2	= H_3PO	+ 3 CH_4	⇒	-10.23	23.11	21.42	19.91	17.30	15.15	13.37	11.87
$HPCH_2$	+ H_2	= H_2PCH_3		⇒	-43.45	12.20	11.47	10.92	10.15	9.63	9.26	8.98
PCH	+ H_2	= $HPCH_2$		⇒	-34.93	16.99	16.37	15.89	15.20	14.72	14.37	14.10
PN	+ H_2	= HPNH		⇒	-19.42	10.01	12.18	13.69	15.55	16.58	17.20	17.59
HPNH	+ H_2	= H_2PNH_2		⇒	-45.32	28.84	25.86	23.76	20.96	19.11	17.79	16.79
H_2PNH_2	+ H_2	= PH_3	+ NH_3	⇒	-10.13	15.91	11.86	8.61	3.82	0.60	-1.70	-3.41
$P(NH_2)_3$	+ 3 H_2	= PH_3	+ 3 NH_3	⇒	-14.26	48.16	35.37	25.31	10.56	0.32	-7.09	-12.65
$P(NC_2H_6)_3$	+ 3 H_2	= PH_3	+ 3 $HN(CH_3)_2$	⇒	-4.26	43.49	30.27	19.72	4.16	-6.52	-14.16	-19.83
P_4	+ 3 O_2	= P_4O_6		⇒	-507.00	-450.11	-407.74	-372.00	-315.47	-273.00	-240.04	-213.77
P_4	+ 5 O_2	= P_4O_0		⇒	-865.78	-769.47	-692.40	-625.74	-516.83	-431.99	-364.24	-309.00
$P(OCH_3)_3$	+ 3 H_2	= PH_3	+ 3 $HOCH_3$	⇒	13.91	156.57	138.93	124.73	103.30	88.09	76.75	68.03
H_3PO_4	+ 3 H_2	= H_3PO	+ 3 H_2O	⇒	43.97	151.91	140.53	130.46	113.57	100.08	89.13	80.12
$OP(OCH_3)_3$	+ 3 H_2	= H_3PO	+ 3 $HOCH_3$	⇒	50.04	134.34	123.72	114.43	98.99	86.76	76.91	68.82
H_3PO_3	+ 2 H_2	= H_3PO	+ 2 H_2O	⇒	31.14	101.01	92.12	84.47	71.98	62.26	54.51	48.22
$OP(OH)_2CH_3$	+ H_2O	= H_3PO_4	+ CH_4	⇒	-15.38	-42.39	-40.38	-38.44	-34.90	-31.86	-29.28	-27.07
H_2PSH	+ H_2	= PH_3	+ H_2S	⇒	-1.51	14.96	12.98	11.51	9.49	8.17	7.25	6.57
H_2PSCH_3	+ H_2	= PH_3	+ $HSCH_3$	⇒	-0.25	15.19	13.18	11.71	9.71	8.43	7.53	6.87
H_3PS_4	+ 4 H_2	= PH_3	+ 4 H_2S	⇒	6.56	43.96	34.01	26.18	14.75	6.87	1.16	-3.17

Table S2 Averaged and normalized ligand binding ernergies in kcal/mol for small-sized clusters.

Complexes	TPSS/def2-SVP	DFTB2/auorg$^{\alpha}$	DFTB2/auorg$^{\alpha\prime}$	DFTB2/auorg$^{\chi\prime}$
$Au_2(PH_3)_2$	-27.8	-39.1	-48.1	-45.9
$[Au_3(PH_3)_3]^+$	-47.8	-60.9	-67.3	-65.4
$Au_4(PH_3)_2$	-33.9	-40.3	-49.5	-47.6
$Au_2(PMe_3)_2$	-36.8	-41.8	-49.9	-47.7
$[Au_3(PMe_3)_3]^+$	-64.5	-66.8	-73.2	-71.4
$Au_4(PMe_3)_2$	-46.4	-44.2	-52.6	-50.8
$Au_2(PPh_3)_2$	-37.9	-41.8	-49.7	-47.6
$[Au_3(PPh_3)_3]^+$	-70.0	-70.0	-77.0	-74.8
$Au_4(PPh_3)_2$	-48.7	-45.0	-53.1	-51.4

Table S3 Averaged and normalized ligand binding ernergies in kcal/mol for moderate-sized clusters.

Complexes	TPSS/def2-SVP	DFTB2/auorg$^{\alpha}$	DFTB2/auorg$^{\alpha\prime}$	DFTB2/auorg$^{\chi\prime}$
$[Au_6(PH_3)_6]^{2+}$	-52.1	-63.1	-69.4	-67.6
$[Au_7(PH_3)_7]^+$	-31.6	-34.5	-43.4	-42.4
$[Au_8(PH_3)_8]^{2+}$	-43.6	-47.9	-55.4	-54.1
$[Au_9(PH_3)_8]^{3+}$	-57.3	-69.2	-75.3	-73.4
$[Au_{11}(PH_3)_{10}]^{3+}$	-51.8	-56.7	-63.5	-62.3
$[Au_{13}(PH_3)_{12}]^{5+}$	-72.2	-84.8	-90.3	-88.5
$[Au_{20}(PH_3)_{16}]^{4+}$	-46.2	-48.6	-56.0	-54.4
$Au_{22}(PH_3)_{12}$	-25.5	-19.4	-30.0	-28.6
$[Au_6(PMe_3)_6]^{2+}$	-71.1	-70.7	-77.3	-75.4
$[Au_7(PMe_3)_7]^+$	-46.5	-39.4	-48.2	-46.9
$[Au_8(PMe_3)_8]^{2+}$	-60.8	-54.9	-62.4	-61.0
$[Au_9(PMe_3)_8]^{3+}$	-79.0	-78.5	-85.0	-83.1
$[Au_{11}(PMe_3)_{10}]^{3+}$	-71.2	-65.2	-72.6	-71.0
$[Au_{13}(PMe_3)_{12}]^{5+}$	-98.2	-97.8	-104.1	-102.3
$[Au_{20}(PMe_3)_{16}]^{4+}$	-65.9	-57.2	-64.6	-62.9
$Au_{22}(PMe_3)_{12}$	-41.8	-24.0	-34.1	-32.9

Table S4 Averaged and normalized ligand binding ernergies in kcal/mol for large-sized clusters, TPSS denotes TPSS/def2-SVP, α denotes DFTB2/auorg$^{\alpha}$, TPSS//α denotes TPSS/def2-SVP//DFTB2/auorg$^{\alpha}$, $\alpha\prime$ denotes DFTB2/auorg$^{\alpha\prime}$, TPSS//$\alpha\prime$ denotes TPSS/def2-SVP//DFTB2/auorg$^{\alpha\prime}$, $\chi\prime$ denotes DFTB2/auorg$^{\chi\prime}$, TPSS//$\chi\prime$ denotes TPSS/def2-SVP//DFTB2/auorg$^{\chi\prime}$.

Complexes	TPSS	α	TPSS//α	$\alpha\prime$	TPSS//$\alpha\prime$	$\chi\prime$	TPSS//$\chi\prime$
$[Au_6(dppp)_4]^{2+}$	-67.6	-58.1	-64.9	-64.9	-64.2	-62.8	-64.5
$[Au_6(PPh_3)_6]^{2+}$	-87.2	-82.2	-86.2	-89.4	-85.9	-87.4	-85.8
$[Au_7(PPh_3)_7]^+$	-63.9	-49.9	-64.6	-58.6	-64.7	-57.6	-63.8
$[Au_8(PPh_3)_7]^{2+}$	-80.6	-73.0	-78.6	-80.2	-79.0	-78.3	-79.0
$[Au_8(PPh_3)_8]^{2+}$	-79.6	-69.7	-79.3	-77.3	-79.3	-75.8	-78.8
$[Au_8S_2(dppm)_4]^{2+}$	-70.0	-61.5	-71.9	-68.8	-72.1	-68.1	-70.4
$[Au_9(PPh_3)_8]^{3+}(C_4)$	-98.9	-92.3	-99.8	-99.4	-100.0	-96.4	-99.3
$[Au_9(PPh_3)_8]^{3+}(D_{2h})$	-99.3	-94.2	-99.4	-101.4	-99.8	-99.4	-99.5
$[Au_{11}(PMePh_2)_{10}]^{3+}$ (C_{3v})	-85.8	-76.4	-87.7	-83.8	-87.6	-82.6	-87.0
$[Au_{11}(PMePh_2)_{10}]^{3+}$ (D_{4d})	-85.6	-76.5	-87.0	-84.0	-87.1	-84.9	-86.6
$[Au_{13}(dppm)_6]^{5+}$	-108.6	-104.9	-109.0	-111.7	-109.2	-109.8	-108.6
$[Au_{20}(PP_3)_4]^{4+}$	-78.4	-57.2	-69.4	-62.6	-70.2	-60.8	-69.5
$Au_{22}(dppo)_6$	-49.5	-30.1	-51.5	-39.1	-50.7	-39.0	-49.5
$[Au_{38}(m\text{-}MBT)_{20}(PPh_3)_4]^{2+}$	-77.8	-58.9	-86.4	-65.5	-86.4	-65.1	-88.1
$Au_{70}S_{20}(PPh_3)_{12}$	-70.5	-47.3	-72.9	-54.8	-72.7	-53.4	-72.8

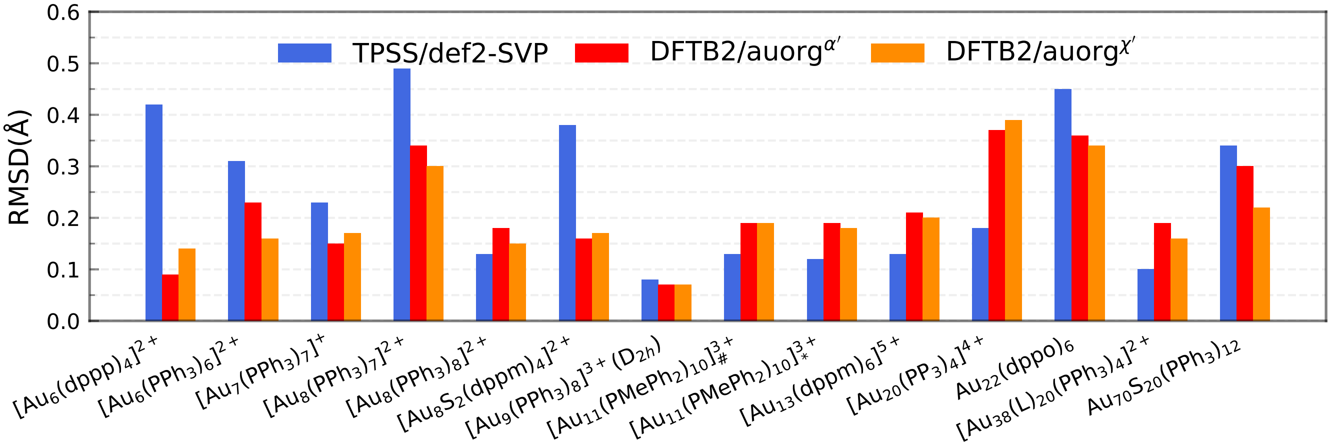

Fig. S2 RMSD over atomic positions for the large-sized phosphine-stabilized gold clusters. The RMSD of atomic positions considers Au, and P atoms for all large-sized phosphine-based gold clusters, $[Au_{11}(PMePh_2)_{10}]^{3+}_{\#}$ denotes $[Au_{11}(PMePh_2)_{10}]^{3+}$ (C_{3v}), $[Au_{11}(PMePh_2)_{10}]^{3+}_{*}$ denotes $[Au_{11}(PMePh_2)_{10}]^{3+}$ (D_{4d}), $[Au_{38}(L)_{20}(PPh_3)_4]^{2+}$ denotes $[Au_{38}(m-MBT)_{20}(PPh_3)_4]^{2+}$.

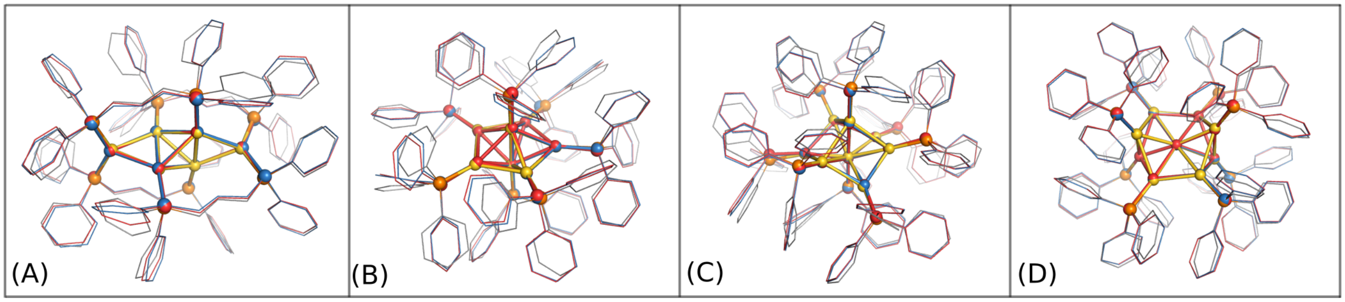

Fig. S3 Overlap of experimental crystal structure (Au in gold, P in orange and C in grey) and optimized DFTB/auorg$^{\alpha}$ and DFTB/auorg$^{\chi'}$ structures. auorg$^{\alpha}$ and auorg$^{\chi'}$ structures are represented by light red and sky blue, respectively. The gold nanoclusters considered in this figure are (A) $[Au_6(dppp)_4]^{2+}$ (BOTSOS), (B) $[Au_7(PPh_3)_7]^{+}$ (BIXZAK), (C) $[Au_8(PPh_3)_8]^{2+}$ (OPAUPF), and (D) $[Au_9(PPh_3)_8]^{3+}$ (MIVPOX-D_{2h}).

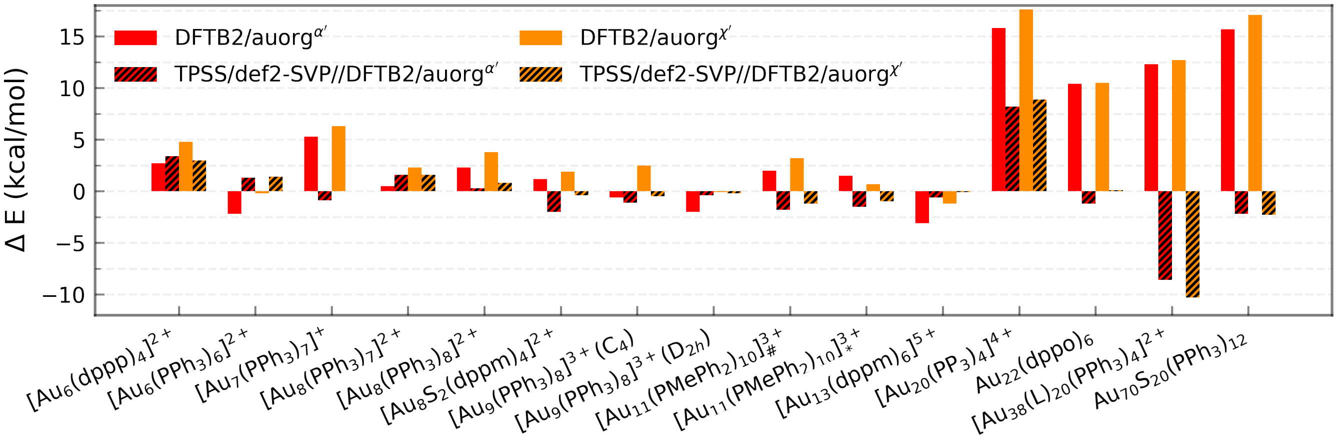

Fig. S4 Deviation in averaged and normalized ligand binding energies for the large-sized phosphine-stabilized gold clusters in reference to the TPSS/def2-SVP binding energies, $[Au_{11}(PMePh_2)_{10}]^{3+}_{\#}$ denotes $[Au_{11}(PMePh_2)_{10}]^{3+}$ (C_{3v}), $[Au_{11}(PMePh_2)_{10}]^{3+}_{*}$ denotes $[Au_{11}(PMePh_2)_{10}]^{3+}$ (D_{4d}), $[Au_{38}(L)_{20}(PPh_3)_4]^{2+}$ denotes $[Au_{38}(m-MBT)_{20}(PPh_3)_4]^{2+}$.

Table S5 Comparison of relative energies with respect to the D_n isomers in kcal/mol as calculated by the following methods for $[Au_9(PPh_3)_8]^{3+}$ and $[Au_{11}(PMePh_2)_{10}]^{3+}$.

Methods	Au_9	Au_{11}
TPSS/def2-SVP	4.98	-3.20
DFTB2/auorg$^{\alpha'}$	11.81	1.78
DFTB2/auorg$^{\alpha}$	11.76	1.38
DFTB2/auorg$^{\chi'}$	10.13	1.82
TPSS/def2-SVP // DFTB2/auorg$^{\alpha'}$	6.29	-5.90
TPSS/def2-SVP // DFTB2/auorg$^{\alpha}$	5.19	-7.63
TPSS/def2-SVP // DFTB2/auorg$^{\chi'}$	6.31	-5.92

Isomerization energy of $[Au_9(PPh_3)_8]^{3+}$ and $[Au_{11}(PMePh_2)_{10}]^{3+}$ clusters

In practice, when studying chemical reactions, absolute cluster binding energies are not as important as relative binding energies, since a ligand is often replaced by another in the same reaction pathway. Therefore, to test the performance of DFTB for the prediction of isomerization energies, two particular examples, namely $[Au_9(PPh_3)_8]^{3+}$ and $[Au_{11}(PMePh_2)_{10}]^{3+}$ were investigated. In the solid phase, $[Au_9(PPh_3)_8]^{3+}$ is reported to have a Au_9 core with D_{2h} symmetry, as the full cluster takes a "butterfly" shape.[3] In a CH_2Cl_2 or methanol solution, this cluster isomerizes to a C_4 core with a crown-like structure.[4] The Au_{11} clusters have idealized C_{3v} and D_{4d} symmetric metal frameworks that differ around three adjacent peripheral sites.[5] The change in the skeletal geometries of Au_{11} clusters is proposed to be caused by the small variations in ligand packing and the presence of anionic ligands coordinated to the C_{3v} Au_{11} gold core. It has been previously predicted that C_4 Au_9 has a 5.7 kcal/mol lower energy than D_{2h} Au_9 in solution,[4] while so far no theoretical predictions were made yet for Au_{11} relative energy isomers. Here, we optimized the isomer structures of Au_9 (D_{2h} and C_4) and Au_{11} (C_{3v} and D_{4d}) with both DFTB and TPSS methods. Their relative structural energies are compared in Figure S5.

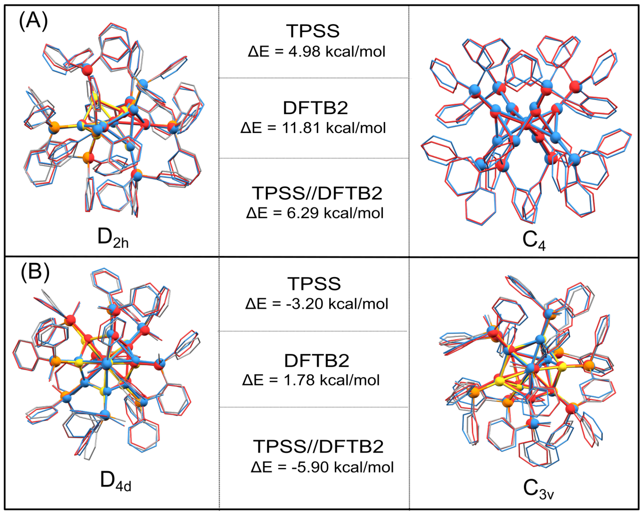

Fig. S5 Overlap of experimental X-ray (Au in gold, P in orange and C in grey) and optimized structures of (A) $[Au_9(PPh_3)_8]^{3+}$ D_{2h} and C_4 isomers and (B) $[Au_{11}(PMePh_2)_{10}]^{3+}$ D_{4d} and C_{3v} isomers. auorg$^{\alpha'}$ and DFT structures are represented by light red and sky blue, respectively. The relative energies in kcal/mol with respect to the D_n isomers are also shown as calculated by TPSS/def-SVP, DFTB2/auorg$^{\alpha'}$ and TPSS/def2-SVP//DFTB2/auorg$^{\alpha'}$. Note that the $[Au_9(PPh_3)_8]^{3+}$ C_4 isomer does not have an available experimental crystal structure.

The relative energies of the isomers presented in Figure S5 were calculated with respect to the D_n isomers (on the left side) as the reference energy. For the $[Au_9(PPh_3)_8]^{3+}$ cluster, all methods predict that the higher symmetry D_{2h} structure is more stable than the less symmetric C_4 structure, with auorg$^{\alpha'}$ overstabilizing the high symmetry by about 5 kcal/mol. In the case of $[Au_{11}(PMePh_2)_{10}]^{3+}$ isomers, the TPSS/def2-SVP calculated C_{3V} isomer is lower in energy than the D_{4d} isomer, while DFTB methods still predict a slightly more stable high symmetry structure. We note that both the isomer relative energies and their DFTB deviations are very small, around 5 kcal/mol, and that it is difficult to achieve perfect agreement even between different density functionals for this energy range. TPSS/def2-SVP single point energy calculations using the DFTB2/auorg$^{\alpha'}$ optimized structures reduce the differences between the relative energies to only 1.33 and 2.72 kcal/mol for Au_9 and Au_{11} isomers, respectively, recovering the isomer energy ordering of the full TPSS

calculations. The complete comparison of the isomer relative energies as calculated by DFTB with all parameter sets is shown in Table S5 in the Supporting Information. The presented comparison demonstrates that, if one wishes to map the potential energy of a certain cluster with many isomers that are only a few kcal/mol apart in relative energies, performing geometry optimizations and pre-optimize structures with the DFTB method is a viable option to save computer time.

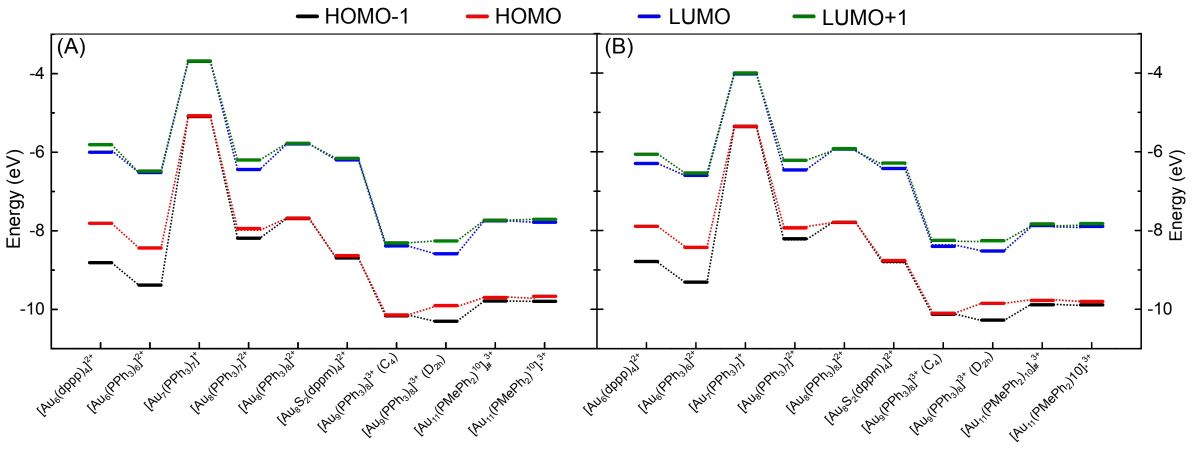

Fig. S6 Energy level diagram for the frontier orbitals of various clusters as calculated by (A) DFTB/auorg$^{\alpha}$ and (B) DFTB2/auorg$^{\chi'}$, $[Au_{11}(PMePh_2)_{10}]^{3+}_{\#}$ denotes $[Au_{11}(PMePh_2)_{10}]^{3+}$ (C_{3v}), $[Au_{11}(PMePh_2)_{10}]^{3+}_{*}$ denotes $[Au_{11}(PMePh_2)_{10}]^{3+}$ (D_{4d}). Dashed lines are included to guide the eye.

Table S6 Comparison of HOMO and LUMO in eV as calculated by DFTB/auorg$^{\alpha'}$, DFTB/auorg$^{\alpha}$, DFTB/auorg$^{\chi'}$, and DFT for small gold clusters.

Complexes	TPSS/def2-SVP	DFTB2/auorg$^{\alpha'}$	DFTB2/auorg$^{\alpha}$	DFTB2/auorg$^{\chi'}$
$[Au_6(dppp)_4]^{2+}$ (BOTSOS)	-7.40 / -5.66	-8.00 / -6.45	-7.81 / -6.00	-7.89 / -6.30
$[Au_6(PPh_3)_6]^{2+}$ (CATPAO10)	-8.15 / -6.22	-8.51 / -6.82	-8.44 / -6.52	-8.43 / -6.60
$[Au_7(PPh_3)_7]^{+}$ (BIXZAK)	-5.10 / -3.47	-5.38 / -4.10	-5.07 / -3.69	-5.35 / -4.02
$[Au_8(PPh_3)_7]^{2+}$ (BASWUN)	-7.70 / -5.99	-8.01 / -6.68	-7.94 / -6.44	-7.93 / -6.47
$[Au_8(PPh_3)_8]^{2+}$ (OPAUPF)	-7.51 / -5.29	-7.83 / -6.18	-7.68 / -5.79	-7.79 / -5.93
$[Au_8S_2(dppm)_4]^{2+}$ (LEVKIJ)	-7.97 / -5.67	-8.86 / -6.52	-8.63 / -6.20	-8.76 / -6.42
$[Au_9(PPh_3)_8]^{3+}$ (MIVPOX-C_4)	-9.84 / -7.94	-10.17 / -8.58	-10.14 / -8.38	-10.10 / -8.40
$[Au_9(PPh_3)_8]^{3+}$ (MIVPOX-D_{2h})	-9.63 / -8.01	-9.92 / -8.74	-9.91 / -8.59	-9.85 / -8.52
$[Au_{11}(PMePh_2)_{10}]^{3+}$ (ZUCMAL)	-9.34 / -7.23	-9.86 / -8.13	-9.70 / -7.75	-9.78 / -7.87
$[Au_{11}(PMePh_2)_{10}]^{3+}$ (ZUCMEP)	-9.43 / -7.23	-9.83 / -8.15	-9.67 / -7.78	-9.8 0 / -7.90
$[Au_{13}(dppm)_6]^{5+}$ (LEVKAB)	-13.34 / -12.28	-13.73 / -12.48	-13.77 / -12.39	-13.69 / -12.40
$[Au_{20}(PP_3)_4]^{4+}$ (POFPUX)	-9.73 / -8.53	-10.28 / -9.346	-10.06 / -9.00	-10.21 / -9.16
$Au_{22}(dppo)_6$ (TOCFIC)	-2.11 / -1.67	-2.75 / -2.00	-2.30 / -1.73	-2.64 / -1.98
$[Au_{38}(m-MBT)_{20}(PPh_3)_4]^{2+}$ (CEMZIG)	-7.19 / -6.07	-7.55 / -6.50	-7.52 / -6.50	-7.52 / -6.40
$Au_{70}S_{20}(PPh_3)_{12}$ (TELMUV)	-3.45 / -2.90	-3.59 / -3.13	-3.47 / -2.99	-3.65 / -3.13

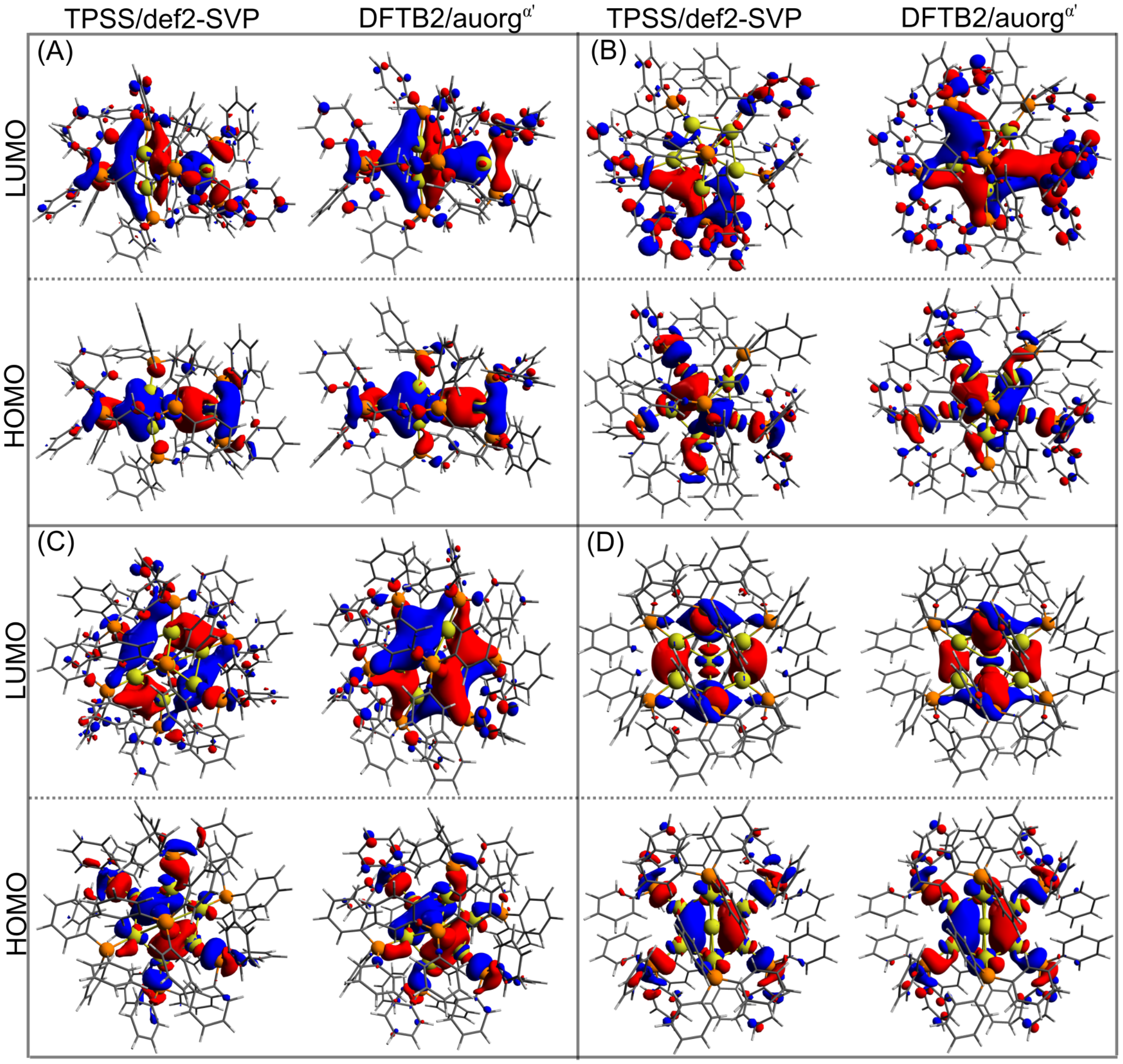

Fig. S7 HOMO and LUMO of (A) $[Au_6(dppp)_4]^{2+}$, (B) $[Au_7(PPh_3)_7]^{+}$(BIXZAK), (C)$[Au_8(PPh_3)_8]^{2+}$ (OPAUPF) and (D) $[Au_9(PPh_3)_8]^{3+}$(MIVPOX-D_{2h}) clusters as calculated by TPSS/def2-SVP and DFTB2/auorg$^{\alpha'}$; isosurface value = 0.02 a.u.

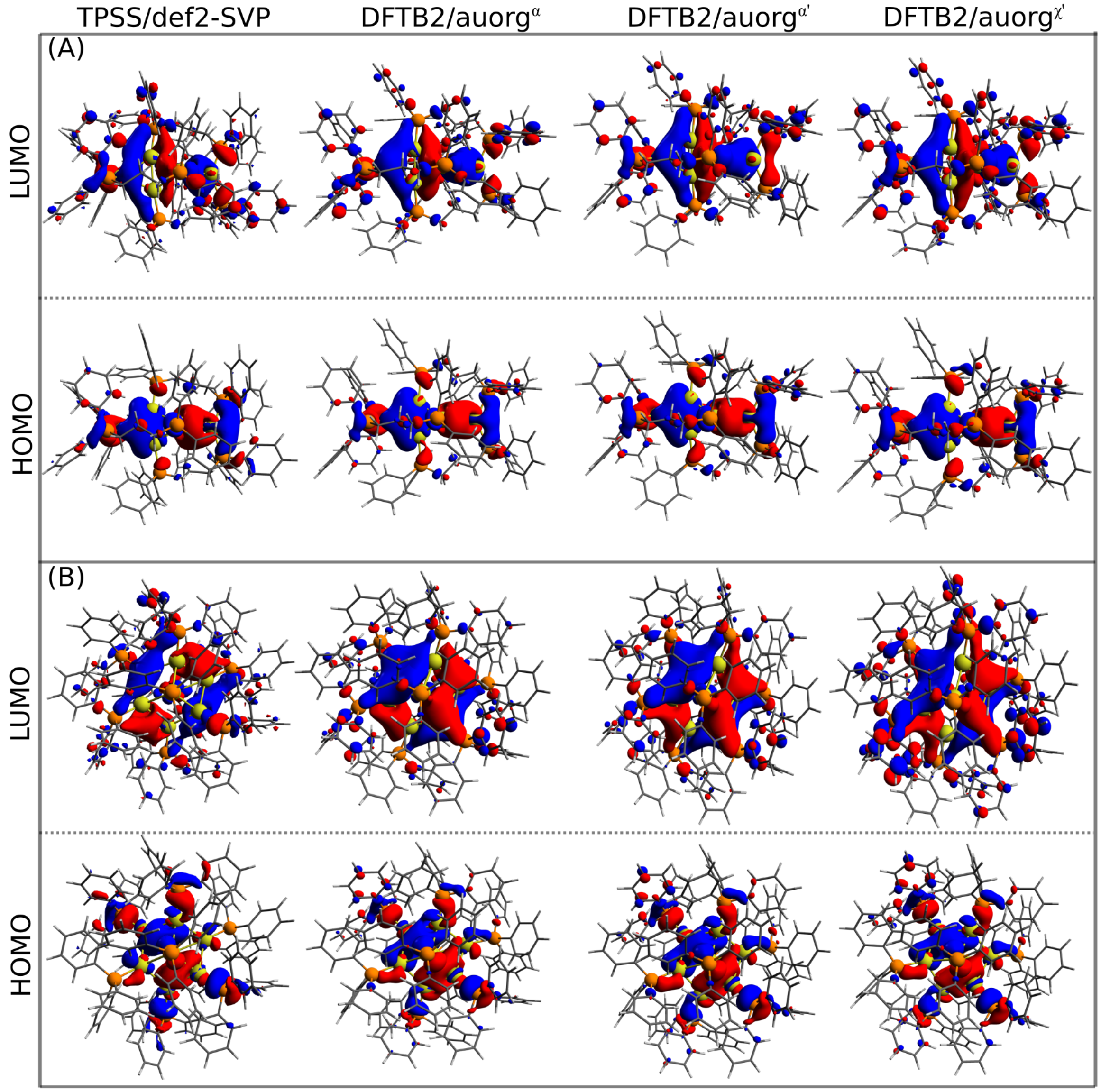

Fig. S8 HOMO and LUMO of (A) $[Au_6(dppp)_4]^{2+}$ and (B) $[Au_8(PPh_3)_8]^{2+}$ (OPAUPF) clusters as calculated by TPSS/def2-SVP, DFTB2/auorg$^{\alpha'}$, DFTB2/auorg$^{\alpha}$, and DFTB2/auorg$^{\chi'}$; isosurface value = 0.02 a.u.

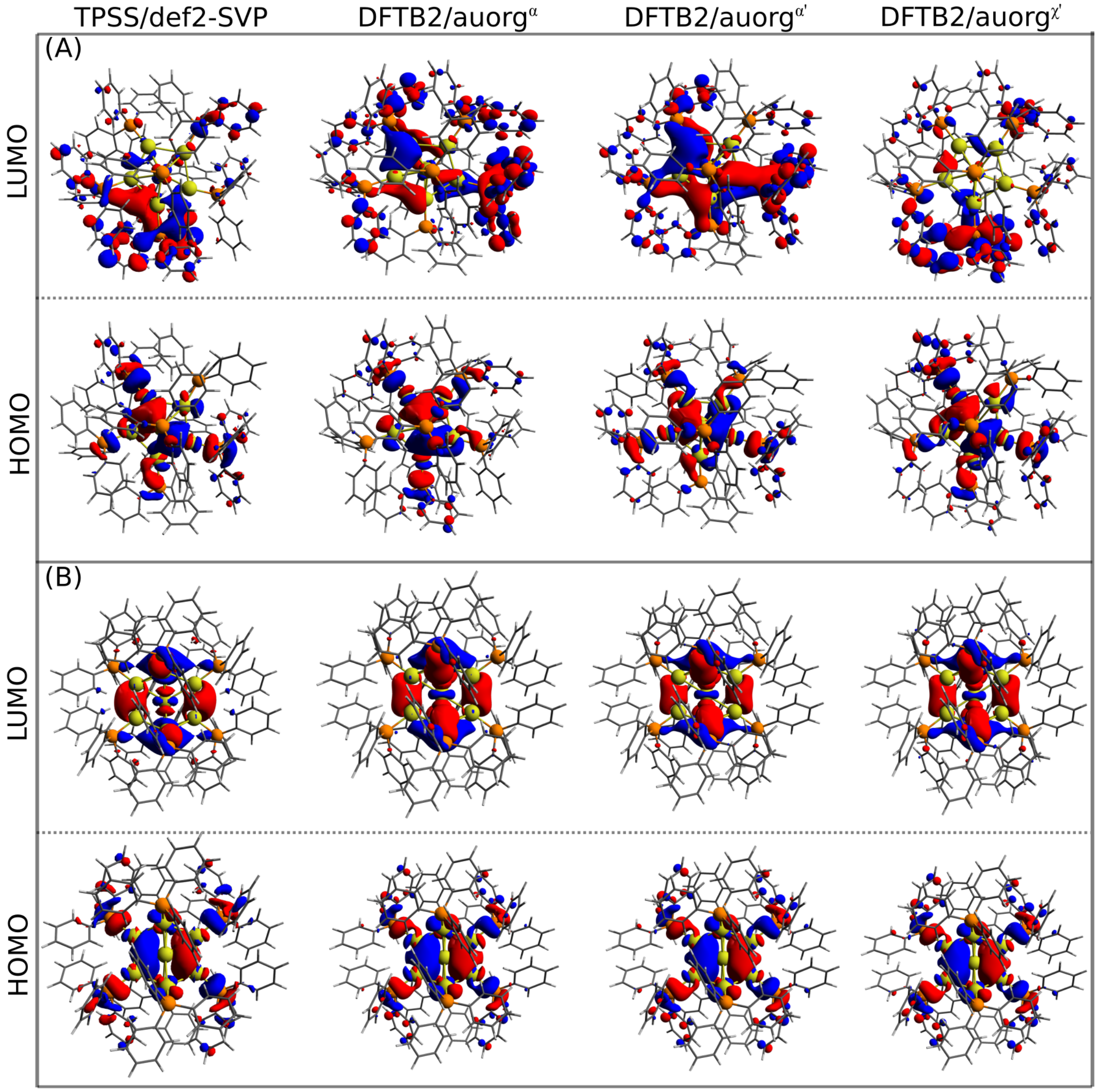

Fig. S9 HOMO and LUMO of (A) $[Au_7(PPh_3)_7]^+$(BIXZAK) and (B) $[Au_9(PPh_3)_8]^{3+}$(MIVPOX-D_{2h}) clusters as calculated by TPSS/def2-SVP, DFTB2/auorg$^{\alpha'}$, DFTB2/auorg$^{\alpha}$, and DFTB2/auorg$^{\chi'}$; isosurface value = 0.02 a.u.

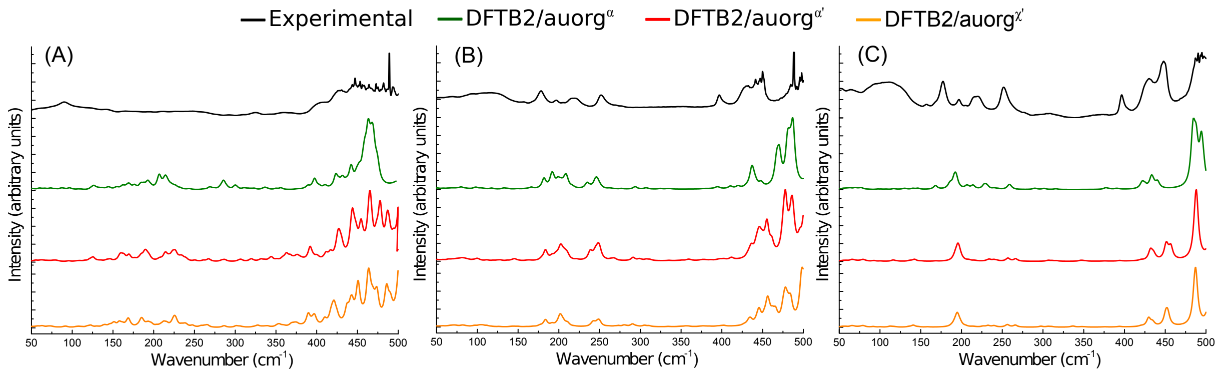

Fig. S10 Experimental (in black), computed auorg$^{\alpha}$ (in green) DFTB2/auorg$^{\alpha'}$ (in red), and auorg$^{\chi'}$ (in orange) far-IR spectra for (A) $[Au_6(dppp)_4]^{2+}$, (B) $[Au_8(PPh_3)_8]^{2+}$, and (C) $[Au_9(PPh_3)_8]^{3+}$ clusters.

Table S7 Summary of contributing transitions as calculated by PBE/def2-SVP, DFTB2/auorg$^{\alpha'}$, DFTB2/auorg$^{\alpha}$ and DFTB2/auorg$^{\chi'}$ for the $[Au_6(dppp)_4]^{2+}$ cluster and their assignment to the experimental spectral peaks, as well as a brief description of the assigned transition modes

Experimental (cm-1)	PBE/def2-SVP (cm^{-1})	Intensity (km/mol)	DFTB2/auorg$^{\alpha'}$ (cm^{-1})	Intensity (km/mol)	DFTB2/auorg$^{\alpha}$ (cm^{-1})	Intensity (km/mol)	DFTB2/auorg$^{\chi'}$ (cm^{-1})	Intensity (km/mol)	Mode description
	85.52	2.6113	80.85	0.008	74.09	0.0142	70.44	0.0102	
90	87.06	3.0854			87.25	87.25	72.85	0.0119	
	89.74	1.7775			96.42	96.42	89.73	0.0102	
	119.65	1.9408	115.45	0.0101	126.85	0.0326	137.82	0.0151	Au core distortion
	125.79	0.6063	121.47	0.0197	130.42	0.0114	140.15	0.0276	
130			125.57	0.053	136.98	0.0149	145.69	0.0247	
					145.9	0.0135			
325	322.41	1.0008	331.99	0.0207	323.49	0.0139	327.67	0.0297	P_2Ph_4–$(CH_3)_3$ wag
	324.42	1.5474	336.12	0.011	336.48	0.0245			
	343.09	2.7216	344.08	0.0424	356.02	0.0225	339.77	0.0165	
	343.54	2.5989	351.05	0.015			351.16	0.0218	
358	346.55	2.2757	358.08	0.0242			353.92	0.0392	P–$(CH_2)_3$–P distortion
	348.18	2.8006	362.86	0.0826			358.28	0.0142	
			367.31	0.0253					
			367.66	0.0208					
	404.31	4.7347	391.89	0.1727	397.37	0.0977	389.66	0.2028	
	404.79	4.6453	395.32	0.0152	397.63	0.0307	394.54	0.0397	
	413.31	0.8692	399.06	0.0149	402.98	0.0236	397.03	0.0523	
410	414.48	13.2331	400.7	0.0301	410.2	0.036	397.37	0.1089	P–C–C–C–P bend
	416.7	9.3675	411.36	0.0713			409.68	0.0942	
	417.66	4.9216	416.86	0.076					
	419.21	6.3026							
	425.22	6.1159	424.99	0.0588	423.33	0.0516	417.41	0.1034	
	427.51	5.486	426.34	0.1706	423.53	0.1058	419.09	0.1037	
431	431.4	3.7808	426.51	0.0396	427.4	0.0423	421.43	0.2731	Au–P stretch and P–Ph stretch
	434.64	7.2679	428.16	0.1126	431.58	0.0984	424.72	0.1076	
	437.65	0.9142	430.5	0.122	436.3	0.0258			
					437.08	0.0141			
	441.92	33.2022	441.98	0.0892	441.62	0.0948	436.94	0.0762	
	442.69	8.5664	443.44	0.0586	442.55	0.1481	437.2	0.164	
			443.85	0.4207			437.2	0.22	
447			447.01	0.1263			441.82	0.1873	
							443.69	0.0253	$P(CH_2)_3Ph_2$ distortion
							447.98	0.5041	
							450.68	0.1313	
							451.31	0.0172	
475	457.58-485.52	Avg. 16.1239	448.67-477.85	Avg. 0.2416	448.97-475.05	Avg. 0.1546	453.31-473.89	Avg. 0.2414	

Table S8 Summary of contributing transitions as calculated by PBE/def2-SVP, DFTB2/auorg$^{\alpha\prime}$, DFTB2/auorg$^{\alpha}$ and DFTB2/auorg$^{\chi\prime}$ for the $[Au_8(PPh_3)_8]^{2+}$ cluster and their assignment to the experimental spectral peaks, as well as a brief description of the assigned transition modes

Experimental (cm-1)	PBE/def2-SVP (cm^{-1})	Intensity (km/mol)	DFTB2/auorg$^{\alpha\prime}$ (cm^{-1})	Intensity (km/mol)	DFTB2/auorg$^{\alpha}$ (cm^{-1})	Intensity (km/mol)	DFTB2/auorg$^{\chi\prime}$ (cm^{-1})	Intensity (km/mol)	Mode description
	173.46	1.4183							
182	176.12	11.457	146.17	0.0229	166.17	0.0347	176.69	0.0247	Au core distortion
	176.23	11.3283							
218	185.35-223.02	Avg. 1.0292	178.65	0.0165	181.34	0.0913	183.25	0.0941	Ph rock
			183.44	0.0782	182	0.1359	183.88	0.089	
			183.68	0.091	191.17	0.23	191.53	0.0338	
			191.34	0.028	192.78	0.0954	191.8	0.0251	
			191.9	0.0272	193.37	0.0533	197.43	0.0213	
			196.17	0.0607	198.77	0.0785	200.69	0.0749	
			199	0.0264	199.03	0.0718	201.22	0.0879	
			201.5	0.0867	202.03	0.0457	201.79	0.0822	
			202.39	0.0949	202.42	0.0446	202.94	0.0779	
			204.11	0.0638	205.02	0.035	204.76	0.0173	
			206.59	0.0143	206.4	0.0268	205.26	0.0602	
			206.82	0.0448	207.82	0.0365	207.56	0.0173	
			208.4	0.0366	208.45	0.0712	210.66	0.0802	
			210.53	0.0729	208.72	0.0708			
					209.04	0.0494			
					209.86	0.0415			
					210.49	0.0333			
					216.17	0.0181			
					217.81	0.0101			
259	247.43-265.45	Avg. 1.3214	221.81	0.0131	234.58	0.1216	240.69	0.0348	Ph rock
			238.05	0.0642	238	0.0263	242.16	0.0414	
			238.66	0.0594	244	0.0378	242.47	0.0303	
			239.42	0.017	244.28	0.0176	243.07	0.0242	
			241.87	0.0138	244.87	0.069	246.36	0.0409	
			242.98	0.0168	245.58	0.0131	248.32	0.0459	
			245	0.061	246.01	0.0521	248.45	0.0297	
			245.74	0.0506	247.41	0.0436	249.57	0.0189	
			248.31	0.1025	247.45	0.0321	249.72	0.0118	
			248.74	0.0422	247.59	0.026	249.88	0.0692	
			248.92	0.028	247.83	0.0218			
			250.29	0.0241	250.49	0.0373			
			250.41	0.0641	256.04	0.0008			
					262.06	0.0116			
398	390.38-406.89	Avg. 0.1938	351.07-402.89	Avg. 0.0035	375.66-396.94	Avg. 0.0033	350.18-402.90	Avg. 0.0038	Ph twist
436	426.97-441.72	Avg. 2.6561	431.28-455.63	Avg. 0.0873	410.22-448.34	Avg. 0.0423	427.38-457.86	Avg. 0.0885	PPh_3 distortion
480	498.85-507.62	Avg. 21.2375	460.98-487.34	Avg. 0.2324	466.34-487.27	Avg. 0.2316	462.09-484.41	Avg. 0.2373	

Table S9 Summary of contributing transitions as calculated by PBE/def2-SVP, DFTB2/auorg$^{\alpha\prime}$, DFTB2/auorg$^{\alpha}$ and DFTB2/auorg$^{\chi\prime}$ for the $[Au_9(PPh_3)_8]^{3+}$ cluster and their assignment to the experimental spectral peaks, as well as a brief description of the assigned transition modes

Experimental (cm-1)	PBE/def2-SVP (cm^{-1})	Intensity (km/mol)	DFTB2/auorg$^{\alpha\prime}$ (cm^{-1})	Intensity (km/mol)	DFTB2/auorg$^{\alpha}$ (cm^{-1})	Intensity (km/mol)	DFTB2/auorg$^{\chi\prime}$ (cm^{-1})	Intensity (km/mol)	Mode description
			136.28	0.0161	129.37	0.0176	141.33	0.0351	
157	148.81	2.2532	142.19	0.0637	131.6	0.0153	147.08	0.017	
			142.82	0.021	139.97	0.0291	158.37	0.0141	
			150.77	0.0136					
	163.28	2.6164							
177	169.95	9.6724	168.16	0.0136	168.01	0.1323	188	0.0321	
	174.7	17.3597							Au core distortion
	186.07	1.5613	190.19	0.0189	190.87	0.0694	194.67	0.2618	
	195	2.025	192.12	0.0159	191.15	0.1564	195.37	0.2421	
	198.75	4.7331	192.73	0.1244	191.84	0.1133	198.78	0.0721	
197	204.53	3.7443	193.1	0.0149	193.45	0.1528	205.06	0.0328	
			195.64	0.2757	193.51	0.1877			
			196.09	0.2851					
			208.95	0.0403					
	214.56	0.6717	215.13	0.0134	206.25	0.0615	213.7	0.0159	
	214.91	0.6573	217.12	0.015	207.4	0.0581	215.86	0.012	
	217.12	2.4079			214.06	0.1124	226.53	0.0412	
220*	217.77	0.1111			214.39	0.0153			Ph rock
	218.61	0.0122							
	218.69	1.4505							
	219.36	1.3855							
	238.93-251.52	Avg. 1.4416	231.09	0.0153	227.83	0.1353	232.29	0.0227	
			233.76	0.0244	229.86	0.0213	232.47	0.0258	
			233.97	0.0239	231.21	0.1022	232.84	0.0239	
			234.03	0.0215	240.05	0.0349	239.67	0.0522	
252			239.78	0.0499	244.58	0.0159	241.13	0.0175	PPh_3 distortion
			243.36	0.029	258.28	0.1192	256.19	0.1159	
			256.9	0.1193	259.19	0.0141	256.39	0.0101	
					259.32	0.0138			
					260.15	0.0371			
397	390.16-401.64	Avg. 0.2980	394.25	0.0091	389.87	0.0188	394.01	0.0114	
			420.43	0.0133			420.46	0.0146	
			431.79	0.401			429.48	0.397	
			432.69	0.0108			430.32	0.0537	
430	424.59-431.16	Avg. 2.4632	432.7	0.0339	412.30-435.88	Avg. 0.074	433.86	0.03	Ph twist
			434.9	0.0315			434.53	0.1603	
			435.54	0.1803			435.16	0.0201	
			435.91	0.0288					
			437.89	0.0106					
448	438.55-444.13	Avg. 5.2400	451.01-456.88	Avg. 0.2211	440.57	0.2226	450.55-453.26	Avg. 0.2197	PPh_3 distortion
480	489.33-492.65	Avg. 18.8798	486.37-488.36	Avg. 0.5519	483.27-496.16	0.5163	485.92-487.96	0.562	

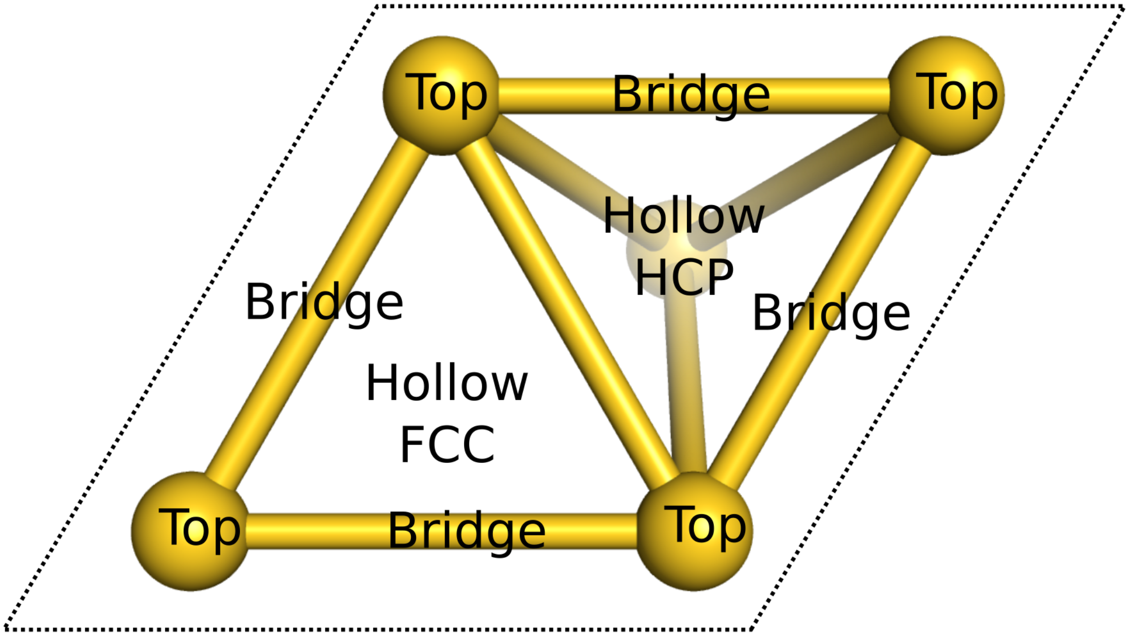

Fig. S11 Different adsorption binding sites on Au (111) surface, only the Au atoms of the top two layers are shown.

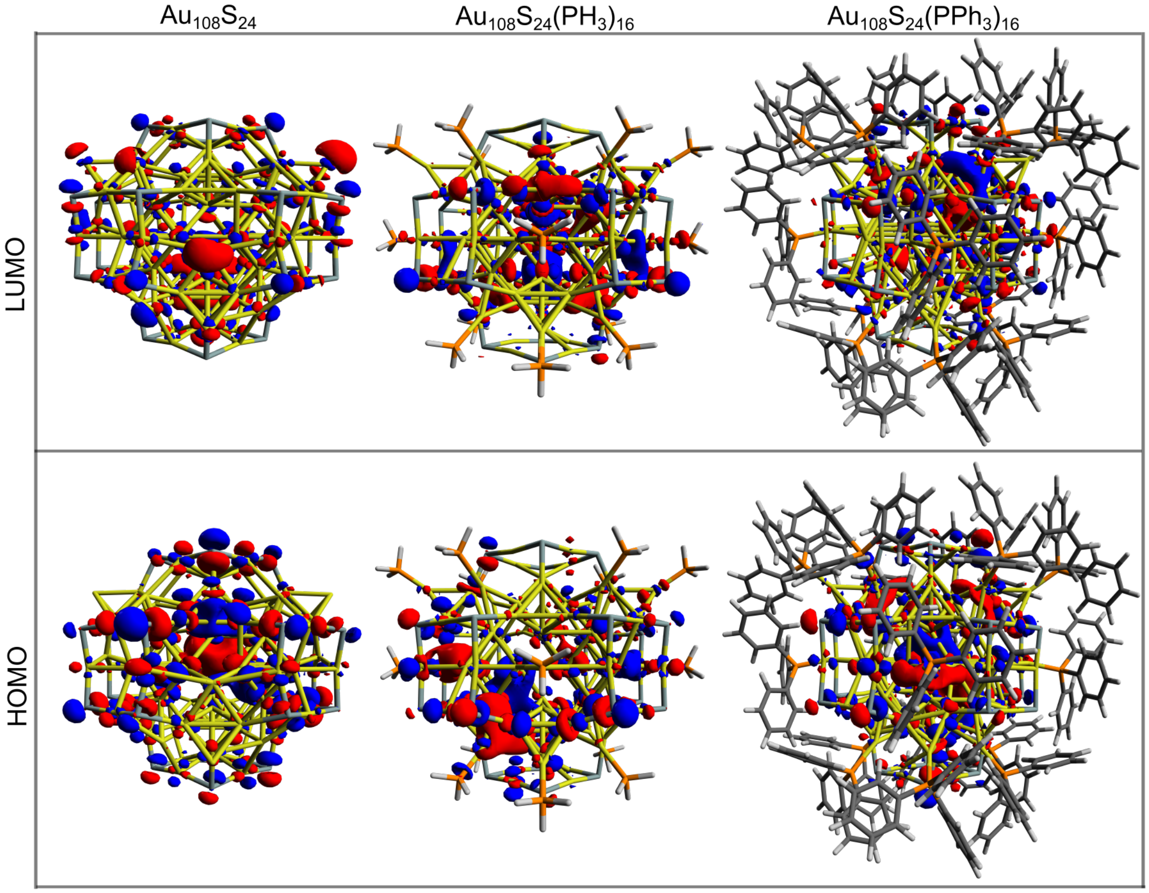

Fig. S12 HOMO and LUMO plots of $Au_{108}S_{24}$, $Au_{108}S_{24}(PH_3)_{16}$, and $Au_{108}S_{24}(PPh_3)_{16}$ clusters as calculated by TPSS/def2-SVP//DFTB2/auorg$^{\alpha\prime}$; isosurface value = 0.015 e$^{0.5/a_0^3}$.

Table S10 Summary of contributing transitions determined by DFTB2/auorg$^{\alpha'}$ for the $[Au_{108}S_{24}(PPh_3)_{16}]$ cluster and brief description of the assigned transition modes, part 1.

DFTB2/auorg$^{\alpha'}$ (cm-1)	Intensity (km/mol)	Mode description
26.387	0.01195	Au core distortion
54.087	0.01243	
54.116	0.01255	
56.459	0.01452	
73.484	0.01721	
76.592	0.0112	
91.121	0.01114	Au core distortion
91.466	0.01238	
93.606	0.01015	asymmetric stretching Au-S-Au (planar rings) and PPh_3 twisting
93.638	0.01133	
102.984	0.01016	Au core distortion
103.05	0.0104	
110.089	0.01135	
113.072	0.01073	
114.583	0.01228	
118.282	0.02662	
118.435	0.02339	
118.772	0.01639	Au-Au-Au rocking (connected to planar rings)
119.34	0.01649	Au-Au-Au scissoring (connected to planar rings)
120.403	0.01194	Au-P asymmetric stretch and Au core distortion
120.684	0.01922	
124.009	0.01762	Au core distortion
124.278	0.01728	
124.451	0.01	
125.952	0.01163	
133.768	0.01069	
141.793	0.01276	
164.734	0.01712	Au-P stretch
165.461	0.01824	
165.477	0.01777	
167.11	0.01346	
167.291	0.01307	Au-Au symmetric stretching (connected to PPh_3)
180.925	0.02166	
181.058	0.01548	Au-P stretch
182.297	0.02001	
182.471	0.03368	
182.914	0.02397	
185.318	0.03596	
185.487	0.02547	
185.676	0.01155	
185.741	0.01826	
185.76-192.27	Avg. 0.0346	Ph rocking
198.523	0.14019	symmetric stretching S-Au-S (planar rings)
200.25	0.10294	
202.781	0.14811	
204.909	0.04854	Ph twist
204.915	0.0242	
205.337	0.04331	
207.877	0.08301	
208.089	0.05917	

Table S11 Summary of contributing transitions determined by DFTB2/auorg$^{\alpha\prime}$ for the $[Au_{108}S_{24}(PPh_3)_{16}]$ cluster and brief description of the assigned transition modes, part 2.

DFTB2/auorg$^{\alpha\prime}$ (cm-1)	Intensity (km/mol)	Mode description
208.161	0.1301	Ph rock
208.402	0.05661	
208.717	0.09316	
209.314	0.05062	
210.34	0.06436	
211.184	0.04972	
218.041	0.01951	S-Au-S rocking (planar rings)
218.41	0.01103	
218.467	0.02623	
218.943	0.11707	Ph rocking
219.181	0.02061	
219.363	0.02627	
219.491	0.04727	
219.741	0.06469	
220.336	0.0208	S-Au-S rocking (planar rings)
222.196	0.04751	
222.776	0.03097	
222.996	0.00298	
223.114	0.04095	
223.41	0.03931	
226.198	0.11918	
234.336	0.00303	
234.5	0.00321	
237.519	0.02785	Ph rock and Au-S stretch
237.597	0.00845	
237.735	0.01358	
237.761	0.02369	
238.013	0.01369	
238.173	0.05096	
238.236	0.05156	
238.412	0.02354	
238.818	0.01568	
238.993	0.02562	
239.258	0.07917	
241.089	0.69303	Au-S stretch
241.192	0.64982	
241.595	0.25861	
241.738	0.01983	
242.066	0.10407	
242.172	0.02482	
243.156	0.05566	
243.74	0.05345	
243.962	0.01056	
247.977	0.06949	
248.71-252.17	Avg. 0.0379	Ph twist
255.09-262.74	Avg. 0.1307	PPh3 distortion and Au-S stretch
265.03-272.86	Avg. 0.0391	Au_4S_4 planar ring rock
274.386	0.05491	Au_4S_4 symmetric stretch
276.211	0.15656	
276.568	0.19116	
277.186	0.11359	Au_4S_4 asymmetric stretch
279.671	0.03883	
279.781	0.08326	

Table S12 Summary of contributing transitions determined by DFTB2/auorg$^{\alpha\prime}$ for the $[Au_{108}S_{24}(PPh_3)_{16}]$ cluster and brief description of the assigned transition modes, part 3.

DFTB2/auorg$^{\alpha\prime}$ (cm-1)	Intensity (km/mol)	Mode description
280.284<br>285.686	0.0261<br>0.06474	Au_4S_4 twist
287.051<br>287.412	0.06944<br>0.40739	Au_4S_4 symmetric stretch
291.177<br>291.437<br>292.643<br>294.434<br>297.485<br>302.74	0.03524<br>0.01303<br>0.02394<br>0.02065<br>0.01415<br>0.02364	PPh_3 distortion
334.01-372.66	Avg. 0.0205	Ph twist
431.06-459.86<br>471.809-495.78	Avg. 0.06291<br>Avg. 0.1485	PPh_3 distortion
506.28-520.33	Avg. 0.4313	PPh_3 distortion and Au-S stretch
521.401<br>522.278<br>522.656<br>525.037<br>525.403<br>525.648	0.12228<br>0.02622<br>0.00953<br>0.11265<br>0.24905<br>0.18726	Au-S stretch
532.85-554.38	Avg. 1.6027	Ph twist and Au-S stretch
559.916<br>570.926<br>571.017<br>571.603	9.29723<br>7.77941<br>7.52268<br>1.64805	Au-P stretch and PPh_3 distortion
613.44-648.58	Avg. 0.1124	PPh_3 distortion

Validation of the DFTB Method in Predicting Infrared Spectra of Gold–Thiolates Clusters

To validate the reliability of the DFTB2 method in simulating IR spectra for gold–thiolates nanoclusters, we compare the DFTB predicted IR spectra to published DFT theoretical IR spectra for $Au_4(SCH_3)_4$, $Au_{18}(SCH_3)_{14}$, and $[Au_{25}(SCH_3)_{18}]^-$ clusters.[6–8] In addition, we evaluate the capability of the DFTB2 method in describing ligand-ligand interactions and the effects of ligands on the structure and vibrational properties of the gold–thiolates clusters. We compare DFTB2 calculated IR to DFT calculated and experimental IR spectra for Au_{18} core cluster with six different types of ligand; $-SCH_3$, $-S-c-C_6H_{11}$, $-SPh$, $-p-MBA$, $-SPhNO_2$, and $-TBBT$.[8] The effects of different ligands on the geometry of the Au_{18} core are compared as well. Figure S13 shows that the DFTB calculated IR spectra are comparable to the corresponding DFT spectra predicted by Tlahuice-Flores in his previous work.[8] Similar to the IR spectra of the phosphine-stabilized $[Au_6(dppp)_4]^{2+}$, $[Au_8(PPh_3)_8]^{2+}$, and $[Au_9(PPh_3)_8]^{3+}$ clusters, the DFTB calculated intensities are significantly lower but the normalized spectral shapes agree with their DFT calculated counterparts.

Figure S14 shows DFTB calculated IR spectra of a Au_{18} core cluster with six different types of ligands. Generally, DFTB calculated IR spectra resemble closely the DFT calculated spectra, but with smaller intensities than the reported ones by ≈20-40 km/mol.[8] DFTB reproduces the change in IR spectra with the change of ligands well in comparison to the DFT calculated IR spectra. In particular, the case of $Au_{18}(-S-c-C_6H_{11})_{14}$ clearly shows that the DFTB IR spectrum agrees extremely well with the experimental spectrum (using the same methodology as for the phosphine-stabilized clusters).[9] Small deviations between DFTB and DFT IR spectra might be caused by different initial geometries for the previously published DFT- and our own DFTB-optimized geometries,[8] and because the geometry optimization of these large and complicated clusters can converge to different local minima. Among these Au_{18} clusters, $Au_{18}(p-MBA)_{14}$ and $Au_{18}(SPhNO_2)_{14}$ clusters have highest intensity in their IR signals particularly for the Au-Au and Au-S stretches in $Au_{18}S_{14}$ core clusters when compared to the rest of the clusters. The increase in these IR intensities can be attributed to the large structural distortions caused by $p-MBA$ and $SPhNO_2$ ligands. Figure S15 shows the distortions of the Au_{18} core cluster caused by these ligands. This is consistent with the previous DFT-based study.[8] These results are very encouraging and suggests that the DFTB method is reliable in predicting the IR spectra for thiolate-protected gold clusters and confirms that it is able to describe the ligand-ligand interactions as well as ligands effects on gold clusters congruent with DFT

predictions.

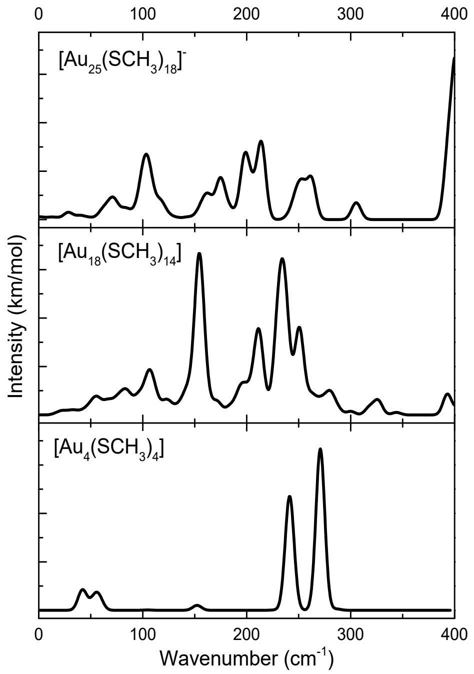

Fig. S13 DFTB calculated IR spectra of $Au_4(SCH_3)_4$, $Au_{18}(SCH_3)_{14}$, and $[Au_{25}(SCH_3)_{18}]^-$.

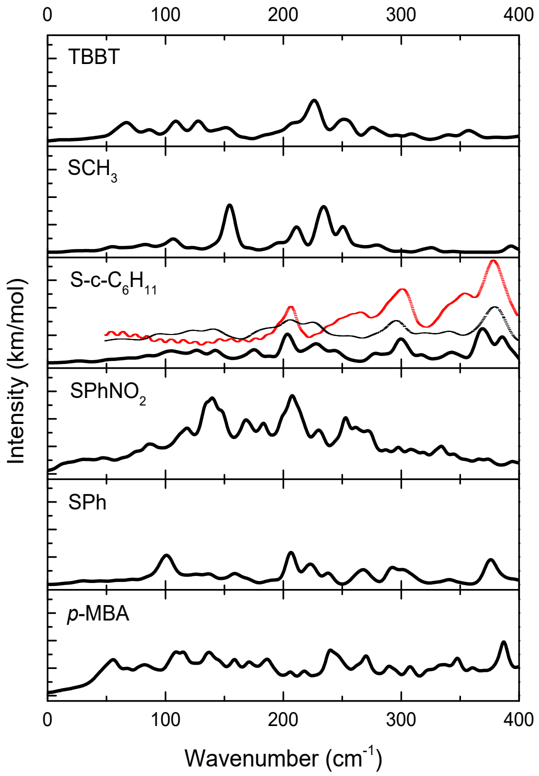

Fig. S14 Calculated IR spectra of $Au_{18}(SCH_3)_{14}$, $Au_{18}(S{-}c{-}C_6H_{11})_{14}$, $Au_{18}(SPh)_{14}$, $Au_{18}(p{-}MBA)_{14}$, $Au_{18}(SPhNO_2)_{14}$, and $Au_{18}(TBBT)_{14}$ clusters using a FWHM of 5 cm^{-1} Gaussian broadening. More intense IR spectra are observed on p−MBA- and $SPhNO_2$-protected clusters. The additional plots in $S{-}c{-}C_6H_{11}$-ligated Au_{18} are the experimental far-IR spectrum (dotted red) and the DFTB-simulated with a wider FWHM of 8 cm^{-1} Gaussian broadening.

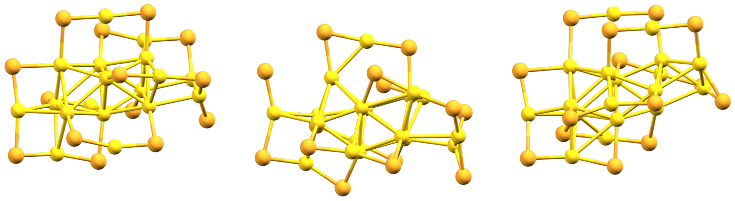

Fig. S15 $Au_{18}S_{14}$ core structures of $Au_{18}(S-c-C_6H_{11})_{14}$, $Au_{18}(p-MBA)_{14}$, and $Au_{18}(SPhNO_2)_{14}$ clusters with four different ligands (ligand structures are omitted for clarity). Au and S atoms are yellow and orange. The clusters are optimized by means of DFTB/auorg$^{\alpha\prime}$ with the D3 dispersion corrections.

Notes and references

1 A. Fihey, C. Hettich, J. Touzeau, F. Maurel, A. Perrier, C. Köhler, B. Aradi and T. Frauenheim, *J. Comput. Chem.*, 2015, **36**, 2075–2087.

2 Y.-S. Lin, G.-D. Li, S.-P. Mao and J.-D. Chai, *J. Chem. Theory Comput.*, 2013, **9**, 263–272.

3 F. Wen, U. Englert, B. Gutrath and U. Simon, *Eur. J. Inorg. Chem.*, 2008, **2008**, 106–111.

4 S. Yamazoe, S. Matsuo, S. Muramatsu, S. Takano, K. Nitta and T. Tsukuda, *Inorg. Chem.*, 2017, **56**, 8319–8325.

5 R. C. B. Copley and D. M. P. Mingos, *J. Chem. Soc., Dalt. Trans.*, 1996, 479–489.

6 A. Tlahuice-Flores, *Mol. Simul.*, 2013, **39**, 428–431.

7 A. Tlahuice-Flores, R. L. Whetten and M. Jose-Yacaman, *J. Phys. Chem. C*, 2013, **117**, 12191–12198.

8 A. Tlahuice-Flores, *Prog. Nat. Sci. Mater. Int.*, 2016, **26**, 510–515.

9 J. F. Alvino, T. Bennett, D. Anderson, B. Donoeva, D. Ovoshchnikov, R. H. Adnan, D. Appadoo, V. Golovko, G. Andersson and G. F. Metha, *RSC Adv.*, 2013, **3**, 22140–22149.
